# Tetrahydrocarbazole as a Versatile Scaffold in Drug Discovery: A Cross-Target SAR Analysis and Design Paradigms

**DOI:** 10.3390/molecules31060977

**Published:** 2026-03-14

**Authors:** Meiling Ma, Shihao Luo, Shaonan An, Zhuang Nie, Zhao Wei, Jiaxuan Zong, Xuanying Li, Chuan Wang, Yuping Tang, Lin Yao

**Affiliations:** 1 Department of Pharmacy, Shaanxi University of Chinese Medicine, Xianyang 712046, China; 2 Department of Medicinal Chemistry and Pharmaceutical Analysis, School of Pharmacy, Fourth Military Medical University, Xi’an 710032, China; 3 State Key Laboratory of Applied Organic Chemistry & Collaborative Innovation Center for Northwestern Chinese Medicine, School of Pharmacy, Lanzhou University, Lanzhou 730000, China

**Keywords:** tetrahydrocarbazole, privileged scaffold, structure-activity relationship (SAR), drug design, medicinal chemistry, cross-target SAR

## Abstract

Tetrahydrocarbazole (THCz) is a privileged scaffold validated by clinically approved drugs such as ondansetron, frovatriptan, and ramatroban and exhibits diverse bioactivities including antimicrobial, antitumor, antidiabetic, and neuroprotective effects. Despite extensive structure–activity relationship (SAR) studies, a systematic integration of findings across different therapeutic targets has been lacking. This review provides a comprehensive SAR dissection of THCz derivatives across key targets (bacterial sliding clamp, BTK, HDAC, AMPK, etc.), analyzing how modifications at key positions of the core scaffold (N-9, C-1, and C-6) influence potency and selectivity. Notably, we highlight four emerging design paradigms: pharmacophore hybridization, conformational constraint, cross-target SAR decoding, and precision intervention. By consolidating fragmented knowledge into a practical cross-target SAR matrix, this review offers a strategic framework for the rational design of next-generation THCz-based therapeutics.

## 1. Introduction

**Figure 1:**
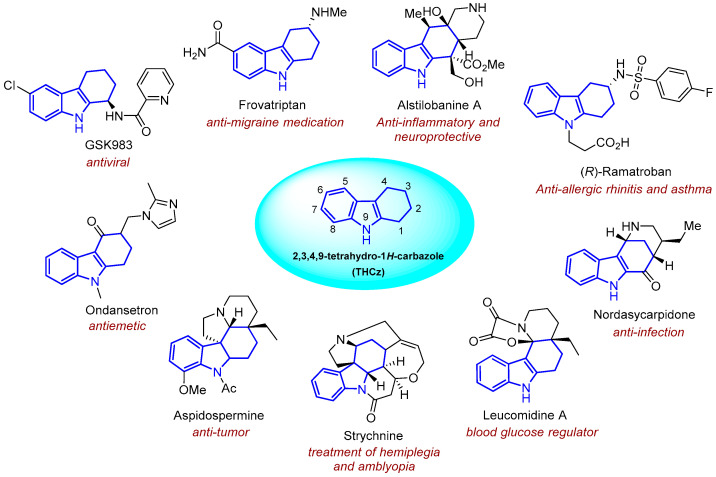
Representative clinical drugs and bioactive agents featuring the THCz scaffold.

Tetrahydrocarbazole (THCz), a rigid and planar tricyclic heterocycle, has emerged as a privileged scaffold in modern drug discovery. Its unique combination of moderate hydrophobicity, extended π-conjugated system, and multiple modifiable positions, such as N-9, C-1, C-2, and C-6, enables precise tuning of electronic properties, lipophilicity, and three-dimensional conformation, facilitating specific interactions with a wide array of biological targets including enzymes, receptors, and nucleic acids [1,2,3]. Naturally occurring THCz derivatives, such as murrayafoline A and ellipticine, are primarily isolated from plants of the Rutaceae, Solanaceae, and Menispermaceae families [4,5]. The clinical relevance of this scaffold is further validated by marketed drugs such as the antiemetic ondansetron [6], the antimigraine agent frovatriptan [7], and the anti-allergic rhinitis drug ramatroban [8] (Figure 1).

THCz derivatives have been shown to exhibit a remarkably broad spectrum of biological activities, including antimicrobial [9,10], antiviral [11], antitumor [12,13], antidiabetic [14,15] and neuroprotective effects [16,17], and has attracted considerable interest across multiple therapeutic areas (Figure 1). While extensive structure–activity relationship (SAR) studies have significantly advanced the potency, selectivity, and pharmacokinetic profiles of THCz-based compounds, most investigations remain confined to single-target paradigms. To date, no comprehensive review has systematically consolidated the research progress on the biological activities of THCz derivatives. Consequently, cross-target SAR patterns, such as the consistent influence of N-9 substitution across structurally and phylogenetically distinct targets like BTK and AMPK, remain poorly delineated. This lack of integrative understanding impedes the formulation of systematic optimization strategies and obscures the identification of broadly applicable design principles, posing significant challenges for rational drug design leveraging the THCz scaffold.

This review addresses this gap by systematically examining the literature from 2000 to 2026 on the biological activities and SAR of THCz derivatives. It should be noted that the analysis is confined to the monomeric scaffold and excludes fused polycyclic and complex alkaloid-like structures, which follow different design logics. Through a comprehensive, site-specific analysis of key modification positions on the THCz skeleton and an extraction of core design strategies, this work distills cross-target SAR trends and delineates practical design paradigms. In this way, we aim to provide a foundational resource to guide future THCz-based therapeutic discovery and development.

## 2. Biological Activities and SAR Studies Targeting Various Biological Targets

### 2.1. Antibacterial Agents

#### 2.1.1. Targeting Bacterial Sliding Clamp

**Figure 2:**
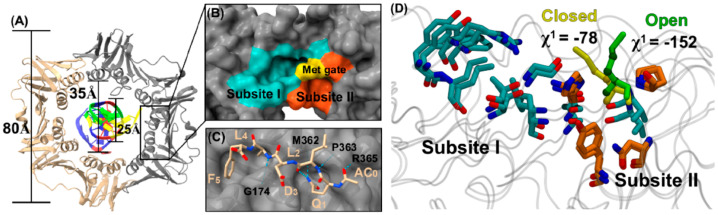
The key structure and subsites of SC. (**A**). X-ray cocrystal structure of the β-SC in complex withDNA, represented in ribbon structure (PDB ID: 3BEP). Each monomer is represented in a different color (gray and cream). Diameters of the β-SC (80 Å), the size of the inner channel (35 Å) and of DNA (25 Å) are shown. A rectangle is used to represent the location of one of the two symmetry related active sites. (**B**) A zoomed-in surface representation of the β-SC active site (PDB ID: 1MMI). Subsite I is colored in teal; Subsite II in orange and the Met gate in yellow. The rest of the β-SC is colored in gray. (**C**) A consensus linear motif (Ac-QLDLF) in the β-SC active site (PDB ID: 4K3Q). The β-SC is colored gray, the carbon backbone of the minimal binding motif is colored in cream with respective labels and the rest is colored in CPK. Hydrogen bonds between the LM and the active site are represented by blue dashed lines with signiffcant residues labeled. (**D**) Superimposed structures of the β-SC to show the Met gate in open and closed conformations. Subsite I, Subsite II and the Met gate are shown in liquorice representation. The rest of the protein is shown in gray (ribbons). The side chain residues forming Subsite I are shown in teal and those forming Subsite II in orange. The open conformation of the Met gate is colored in green (PDB ID: 1OK7) 14 and the closed conformation is shown in yellow (PDB ID: 1MMI). 16 χ1 angles are shown as −152° and −78° for open and closed conformations, respectively with darker colored atoms (dark green and yellow) representing those that form up χ1 angles [18].

The bacterial sliding clamp (SC) is a ring-shaped homodimeric protein that encircles DNA and serves as a mobile platform to recruit DNA replication and repair enzymes. A conserved hydrophobic pocket on the SC surface recognizes short linear motifs (LMs) of partner proteins through two subsites: a deep hydrophobic subsite I that anchors the LM C-terminus, and a shallower subsite II near the pocket entrance. Residue Met362 acts as a dynamic gate closed in the unbound state; it rotates open upon N-terminal engagement, creating a continuous groove that enables stable C-terminal docking (Figure 2). The high conservation of SC across bacteria and its structural divergence from the eukaryotic counterpart PCNA make it an attractive selective antibacterial target [18].

**Figure 3:**
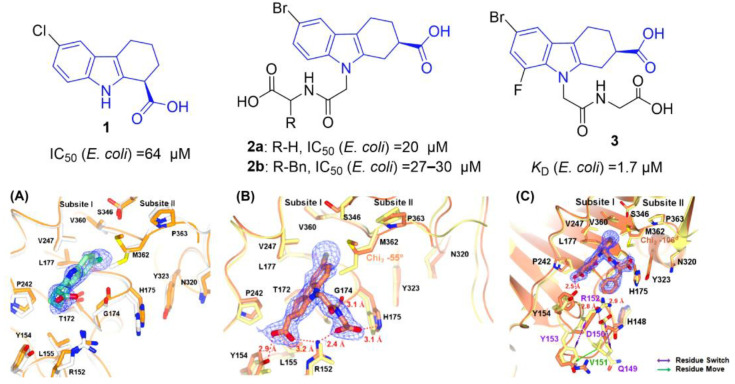
Development of THCz derivatives as the bacterial SC inhibitors. (**A**−**C**) Binding mode of compound **1**, **2a** and **2b** with *E. coli* SC.

Based on their aforementioned research [19], Oakley and coworkers performed a fragment-based screen and identified 6-chloro-THCz (compound **1**) as the highest-affinity ligand for *E. coli* SC (*K*ᵢ = 64 μM) [14]. Co-crystal structures revealed that the THCz core anchors deeply in subsite I, with the 6-chloro substituent forming a halogen bond with Val342 and hydrophobic contacts with Val345. The C-2 carboxylate, required in the (*R*)-configuration, engages in a polar interaction with Arg374. The N-H of the carbazole is not involved in hydrogen bonding, but the N-substituent projects toward subsite II. Introduced an acetamido extension (compound **2a**) created a key hydrogen bond with Gly174 and improved affinity to *K*ᵢ = 11 μM. Further extension with a phenylalanine moiety (compound **2b**) induced an unexpected conformational rearrangement of the H148-Y154 loop, generating a ligand-induced “site III” pocket [20] (Figure 3).

Hoff and colleagues employed nuclear magnetic resonance (NMR) spectroscopy to evaluate a series of THCz derivatives with varied C-6 substituents against the *E. coli* β-clamp [21]. Ligand-observed NMR methods revealed potent binding for bromo- and trifluoromethyl-substituted analogs, with fluoro-substituted variants showing weaker affinity. Isothermal titration calorimetry (ITC) quantified affinities for fluorinated lead **3**. Critically, a ^19^F NMR T_2_-based competition assay was established as a label-free platform for identifying new β-clamp inhibitors (Figure 3).

**Figure 4:**
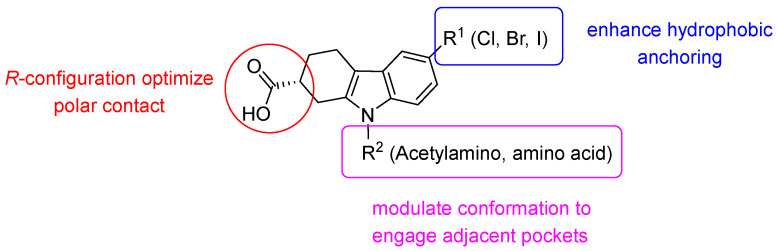
SAR of THCz derivatives as the bacterial SC inhibitors.

Overall, the above studies validated THCz as bacterial SC inhibitors via sequential binding: core anchoring in subsite I, N-substituent-mediated Met362 gating, and multi-pocket synergy. Key SAR principles emerged: C-6 halogen hierarchy (Br/Cl > I > F > H); C-2 stereochemistry critical ((*R*)-enantiomer 20-fold more active); and N-substituents require flexible 2-3 atom linkers to reach subsite II. Electron-donating groups or rigid/bulky N-substituents abolish activity (Figure 4). These insights, including ligand-induced site III formation, provide a clear blueprint for designing potent SC inhibitors and multitarget agents.

**Figure 5:**
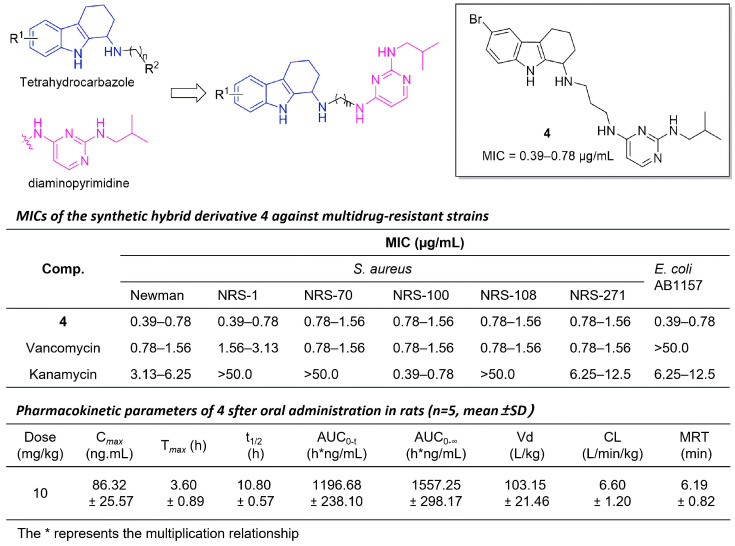
THCz-2,4-diaminopyrimidine hybrids with broad-spectrum antibacterial activity.

In 2019, Chen’s group rationally designed a novel hybrid series by combining the bioactive THCz scaffold with the antibacterial 2,4-diaminopyrimidine pharmacophore, yielding agents with nanomolar activity against multidrug-resistant strains [22]. Amongst them, compound **4** (6-Br, n = 3) exhibited MIC values of 0.39–0.78 µg/mL against MRSA, comparable or superior to vancomycin. Compound **4** showed moderate oral absorption (C_max_ 86 ng/mL at 3.6 h), long half-life (10.8 h), wide tissue distribution, but low systemic exposure, suggesting need for hydrophilicity optimization (Figure 5). SAR analysis revealed that a three-carbon linker between pharmacophores was optimal and that introducing an electron-withdrawing group at the C-6 position of the THCz core significantly enhanced potency. This work validates the hybrid dual-pharmacophore strategy as a viable approach to overcoming bacterial resistance.

#### 2.1.2. Targeting Bacterial MreB

MreB is a prokaryotic actin homolog that polymerizes into dynamic, ATP-dependent filaments at the inner membrane. It maintains cell shape, regulates cell wall synthesis, and assists in chromosome segregation. MreB is conserved across Gram-negative pathogens, absent in humans, and not targeted by existing antibiotics, making it a high-priority antibacterial target with low resistance predisposition [23,24].

MreB inhibitors have evolved through three generations. First-generation benzylisothiouronium derivatives (e.g., compound **5**) induced spherical cell morphology but suffered from poor Gram-negative coverage, solubility, and toxicity [25]. Second-generation inhibitors introduced a rigid THCz core with an amino side chain; lead compound **6** showed 32-fold improved activity against efflux-deficient *P. aeruginosa* (MIC = 1 μg/mL) but remained inactive against wild-type strains [26]. This work established the “THCz core with amino side chain” as a foundational pharmacophore for MreB inhibition.

**Figure 6:**
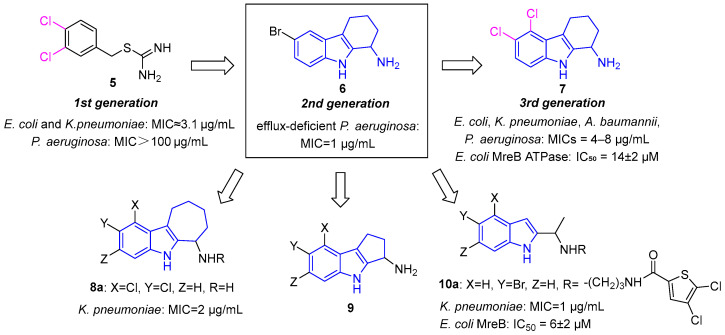
Optimization of the THCz scaffold for MreB Inhibition.

Building on previous designs, LaVoie’s group developed the third-generation inhibitors by hybridizing the dichlorophenyl moiety with the optimized THCz-cyclohexylamine core [12]. Compound **7** achieved broad-spectrum activity (MIC = 4–8 μg/mL against *E. coli*, *K. pneumoniae*, *A. baumannii*, and *P. aeruginosa*), 32-fold improved MreB ATPase inhibition (IC_50_ = 14 ± 2 μM), and low resistance frequency (<10^−9^). Expanding the core to a cycloheptane-fused indole (**8a**) improved potency against *K. pneumoniae* (MIC = 2 μg/mL). Replacing the cyclohexylamine side chain with flexible open-chain analogs and introducing acylation at the terminal amine yielded compound **10a**, the most potent THCz-based MreB inhibitor reported (IC_50_ = 6 ± 2 μM; MIC = 1 μg/mL against *K. pneumoniae*) [27] (Figure 6). This systematic SAR provides a clear framework for further development of MreB inhibitors.

#### 2.1.3. Other Antibacterial Agents

Beyond SC and MreB, THCz derivatives have been identified with other antibacterial mechanisms, including cell wall synthesis inhibition, DNA biosynthesis disruption, virulence modulation, and multi-target effects (Table 1).

The bacterial cell wall is an established antibacterial target due to its essentiality and absence in humans [28,29]. Mellroth and colleagues identified compound **11** through a whole-cell screen against *S. pneumoniae* [30]. SAR revealed that a protonated diamine moiety enables electrostatic interaction with undecaprenyl pyrophosphate (C_55_-PP), a conserved lipid carrier in cell wall biosynthesis [31], while a hydrophobic R^2^ group anchors the compound in the membrane.

Ivanenkova et al. used the dual-fluorescence reporter system pDualrep2 to screen ~47,000 compounds and identified 16 active THCz derivatives [32]. Among these, compound **12** exhibited moderate activity against *E. coli* (MIC = 21 μg/mL) with a high selectivity index (SI > 333), indicating favorable cellular safety. Fluorescence analysis confirmed DNA biosynthesis inhibition as the primary mechanism.

Fortney et al. identified 2,3,4,9-tetrahydro-1H-carbazol-1-amine derivatives that activate the CpxRA two-component system, suppressing uropathogenic *E. coli* (UPEC) virulence without affecting bacterial growth [33,34,35,36]. Fluorination at C-6 markedly improved activity, with compound **13** (EC_50_ = 1.1 μM) achieving 7-fold improvement over the 6-nitro lead.

Gehrmann et al. synthesized bisindole-substituted THCz derivatives and evaluated their antibacterial activity [37]. 5-cyano-substituted *cis*-THCz analogs (e.g., compound **14**) demonstrated promising anti-MRSA activity, outperforming ofloxacin and cefoxitin in some assays.

**Table 1:** THCz-based antibacterial agents with diverse mechanisms.

Core Structure	Key Features	Target/ Mechanism	Representative Compound and Activity	SAR Highlights	Ref.
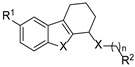	Protonated diamine, hydrophobic R^2^	C_55_-PP (cell wall)	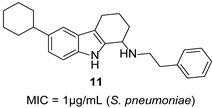	Diamine essential for electrostatic interaction; flexible linker ≥ 2 carbons	[30]
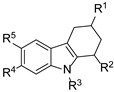	Amino/methylamino at R^2^	DNA biosynthesis	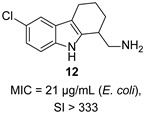	Small R^2^ groups favorable; pyridine at R^3^ improves activity	[32]
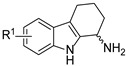	6-F, chiral at C-1 position	CpxRA activation (virulence)	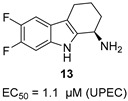	Fluorination enhances potency; (*R*)-enantiomer superior	[36]
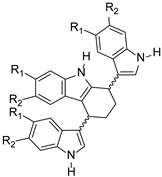	5-cyano, *cis*-configuration	Unknown	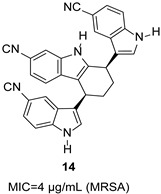	5-Cl or 5-CN optimal; *cis* configuration is better than aromatic carbazole	[37]

#### 2.1.4. Cross-Target SAR for Antibacterial THCz Derivatives

As summarized in Table 2, this systematic SAR analysis reveals that despite the diversity of antibacterial mechanisms targeted by THCz derivatives, common design principles have emerged. Universally, C-6 halogenation enhances potency; stereochemistry at C-1 or C-2 requires strict control; and linker optimization in hybrid designs is critical for achieving dual-target synergy. These cross-target insights provide a practical framework for the rational design of next-generation THCz-based antibacterial agents.

### 2.2. Anti-Fungal Agents

Invasive Fungal Infections (IFIs) pose a serious global health threat, especially to immunocompromised patients [38]. The fungal-specific plasma membrane proton pump (H^+^-ATPase, Pma1) is a promising antifungal target due to its essential role in maintaining membrane potential and nutrient uptake, coupled with the absence of a direct mammalian homolog [39]. However, cross-inhibition of structurally related mammalian P-type ATPases, such as SERCA and Na^+^, K^+^-ATPase, by Pma1-targeting compounds raises potential toxicity concerns, underscoring the critical need for developing highly selective inhibitors [40].

In 2018, Winther’s group identified THCz-based Pma1 inhibitors **15** with potent antifungal activity (IC_50_ = 2–9 μM) [41]. A co-crystal structure with SERCA confirmed the chiral center’s role in target binding. SAR revealed an NH-CH_2_-linker at R^1^ as optimal for potency. Extending R^1^ to biphenyl (**15d**–**e**) retained Pma1 inhibition while reducing activity against mammalian ATPases, leveraging Pma1’s larger binding pocket to enhance fungal selectivity. R^2^ required H; methylation abolished activity via steric clash. R^4^ tolerated Cl, Br, or CF_3_O, enabling physicochemical tuning. Compounds **15** showed broad fungistatic activity against pathogenic Candida species, including *C. albicans*, *C. krusei*, and *C. glabrata*, with MICs of 7.5–21 μM (Table 3), outperforming fluconazole against several strains. Future optimization should exploit structural insights to refine R^1^ substituents and improve PK/toxicity profiles.

**Figure 7:**
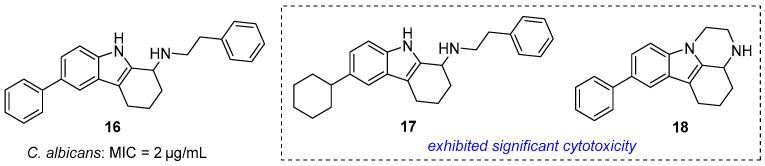
THCz compounds as antifungal agents against *C. albicans*.

Lv et al. identified antifungal THCz **16** via phenotypic screening (MIC = 2 μg/mL vs. *C. albicans*) [42]. At 16 μg/mL, it eradicated the pathogen within 3 h, suppressed biofilm formation, and reduced fungal burden in *Galleria mellonella* (10–20 mg/kg). SAR insights: the tricyclic core is essential, and the specific substituent pattern of **16** confers optimal potency, whereas analogs **17**–**18** showed cytotoxicity, identifying toxicophores to avoid (Figure 7). Mechanistically, **16** induces endoplasmic reticulum stress, offering a novel antifungal strategy.

**Figure 8:**
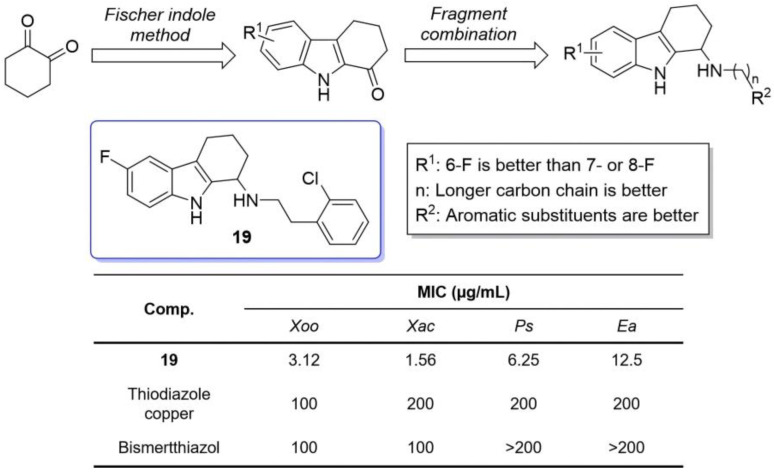
Promising antibacterial and antifungal THCz derivatives against plant pathogens.

In the same year, Liu et al. synthesized and modified THCz derivatives at positions 1 and 6, generating compounds active against plant pathogens [43]. Compound **19** showed the strongest broad-spectrum activity, particularly against *Xanthomonas oryzae* (MIC = 3.12 μg/mL), outperforming thiodiazole copper. In vivo, **19** effectively protected rice from bacterial leaf blight. SAR analysis indicated that electron-withdrawing groups (especially fluoro) at the 6-position significantly enhanced activity, whereas fluorination at the 7- or 8-position reduced it; longer carbon chains at R^2^ and aromatic amines (e.g., p- or o-chloroaniline) also improved potency, while strongly basic amines diminished activity (Figure 8). Mechanistic studies revealed that **19** acts through multi-target mode-disrupting virulence factors and induces oxidative stress-rather than conventional growth inhibition, reducing the likelihood of resistance and highlighting its utility in controlling phytopathogens.

### 2.3. Antiviral Agent

#### 2.3.1. Direct-Acting Antiviral Agents

The NS5B RNA-dependent RNA polymerase (RdRp) is a core enzyme in the HCV replication cycle, responsible for viral RNA synthesis. As it lacks a functional human homolog, NS5B is an attractive target for antiviral drug development, with small-molecule inhibitors considered a promising therapeutic strategy [43].

**Figure 9:**
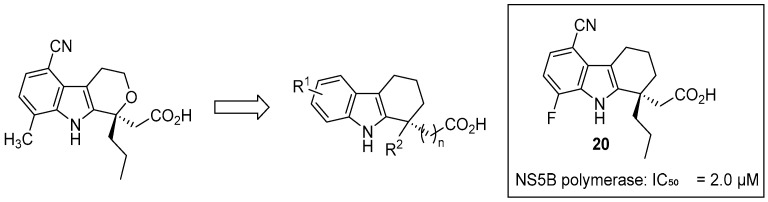
THCz derivatives targeting HCV NS5B polymerase.

In 2006, Gopalsamy and colleagues employed a scaffold-hopping strategy to develop THCz-based NS5B inhibitors from a pyrano[3,4-b]indole lead [44]. Extensive SAR identified critical structural elements: a moderate-length alkyl chain at C-1—particularly *n*-propyl—optimally occupied a hydrophobic pocket in the enzyme, while disubstitution at the 5- and 8-positions—particularly with dichloro or cyano groups—was essential for potent allosteric inhibition, likely due to strong hydrophobic interactions and potential halogen bonding within NS5B’s allosteric pocket. These efforts yielded lead compound **20**, which demonstrated nanomolar enzymatic activity (IC_50_ = 2.0 μM), validating the THCz scaffold as a promising NS5B inhibitor (Figure 9). Although its development was limited by poor cellular activity and a narrow therapeutic window, the compound provided key design insights and proof of concept for targeting viral allosteric pockets, highlighting the scaffold’s potential for broader antiviral applications against HCV and related RNA viruses.

#### 2.3.2. Host-Targeting Antiviral

Human papillomavirus (HPV) is the most prevalent sexually transmitted infection globally and is a major cause of benign and malignant epithelial tumors [45]. Existing treatments remove lesions but do not eliminate the virus, creating an urgent need for direct-acting antiviral drugs.

**Figure 10:**
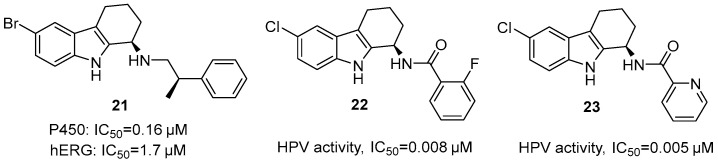
THCz derivatives with potent anti-HPV activity.

Gudmundsson et al. identified a series of 1-amino THCz compounds with potent anti-HPV activity through high-throughput screening [46]. SAR studies revealed that a lipophilic electron-withdrawing group—particularly bromine—at the C-6 position was critical for potency (IC_50_ = 0.03 μM in W12 cells); relocation to C-7 or C-8 sharply reduced activity. At the C-1 amino position, aromatic alkylamino groups—especially those with α-substitution—significantly enhanced activity. Stereochemistry proved decisive: the (*R*)-enantiomer exhibited strong antiviral effects, while the (*S*)-enantiomer was nearly inactive. Compound **21**, bearing an (*R*)-α-methylbenzylamine substituent, potently inhibited HPV-16 DNA replication with minimal cytotoxicity, offering an excellent therapeutic window. However, it showed off-target inhibition of cytochrome P450 (IC_50_ = 0.16 μM) and hERG (IC_50_ = 1.7 μM), attributed to the charged amine and heteroaromatic moieties. Replacement of the 1-amino group with amides revealed that simple acetamides were weak, but lipophilic benzamides restored potent activity (IC_50_ = 0.03 μM). Further optimization identified ortho-fluorobenzamide **22** and 2-pyridinecarboxamide **23** as the most potent analogs, with the (1*R*) configuration again proving essential. Compound **23** exhibited favorable pharmacokinetics in multiple species and may act indirectly by activating host innate immunity rather than directly targeting viral proteins, offering a novel mechanistic direction for anti-HPV drug development [47] (Figure 10).

Subsequent work by Sethna et al. further elucidated the mechanism and selectivity of compound **23** (GSK983) [11]. While these compounds potently inhibit HPV-infected cells, they show negligible toxicity in uninfected primary cells. Cross-line comparisons confirmed high selectivity, likely mediated through modulation of host cell signaling specifically, by inducing interferon-stimulated gene expression to suppress viral replication and gene expression.

Following the above paradigm, Basler and colleagues identified a class of amino-THCz compounds structurally similar to GSK983, which potently inhibit Ebola virus replication [48]. Their mechanism involves targeting host cell dihydroorotate dehydrogenase (DHODH), a key enzyme in de novo pyrimidine synthesis. SAR analysis confirmed that the THCz core and its specific amino side chain are essential for antiviral activity. Comparative evaluation of GSK983’s racemate and enantiomers revealed that both the racemate and the (*R*)-enantiomer exhibited potent nanomolar activity against EBOV minigenome replication and viral proliferation (IC_50_ ≈ 0.02 μM), while the (*S*)-enantiomer was significantly less active (IC_50_ > 1.7 μM). This stereochemical preference aligns with earlier findings for HPV inhibitors, reinforcing that the (*R*)-configuration is critical for antiviral efficacy.

### 2.4. Antitumor Agents

#### 2.4.1. Targeting the Cytoskeleton and Mitotic Progression

Microtubules, dynamic cytoskeletal structures composed of α/β-tubulin heterodimers, play essential regulatory roles in mitosis, maintenance of cellular morphology, and intracellular transport. Due to their critical function in cell division, microtubules represent a well-established target in antitumor drug development [49]. THCz derivatives have shown promising activity in inhibiting microtubule polymerization.

**Figure 11:**
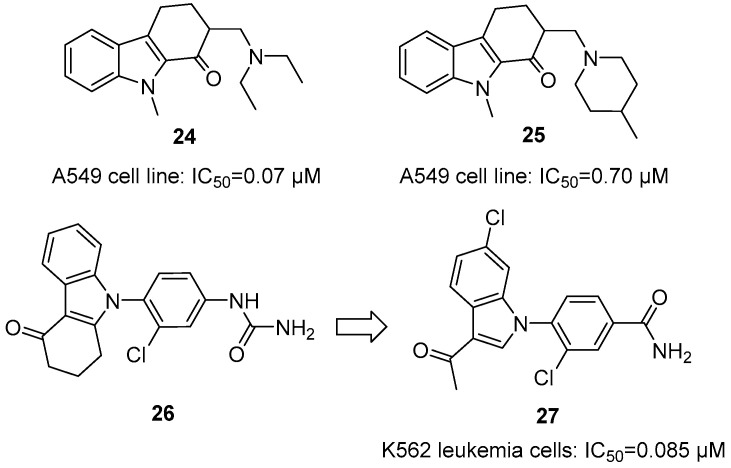
THCz compounds targeting microtubules.

In 2009, Chen et al. designed and synthesized a series of novel 2-aminomethyl-substituted derivatives based on the bioactive THCz scaffold by introducing Mannich base moieties at the C-2 position [50]. In vitro antitumor activity screening revealed that most derivatives exhibited moderate to strong inhibitory effects across several human tumor cell lines, with particularly notable activity against HCT116 colon cancer cells. Among them, compound **24** showed the strongest potency against A549 non-small cell lung cancer cells, with an IC_50_ of 0.07 μM—approximately 35-fold lower than that of the positive control paclitaxel. Compound **25** also displayed promising activity, with an IC_50_ of 0.70 μM (Figure 11). SAR analysis indicated that introducing a methyl group at the N-9 position generally enhanced antitumor activity; within the Mannich base moiety, the diethylamino and 4-methylpiperidyl groups were identified as favorable pharmacophores. Preliminary mechanistic studies showed that compound **24** effectively inhibited tubulin polymerization, suggesting disruption of microtubule dynamics and subsequent induction of tumor cell apoptosis.

Barta et al. serendipitously identified carbazole **26** as a colchicine-site tubulin inhibitor during HSP90 screening [51]. Systematic SAR analysis revealed the following: N-9 amide/urea is essential; substituents adjacent to amide are generally detrimental (except small amines like allylamine); and hydrophobic groups (-Cl, -Br, -OMe, -CF_3_) significantly enhanced activity. Notably, replacing the rigid carbazole with flexible acyl-indole retained potency, demonstrating rigidity unnecessary. Optimization yielded **27** with IC_50_ of 0.085 nM in K562 cells—200-fold more potent than colchicine—establishing a high-quality lead for antitubulin agents (Figure 11).

#### 2.4.2. Targeting DNA Damage and Oxidative Stress

DNA cleavage and free radical-mediated oxidative damage represent another crucial class of antitumor mechanisms. THCz derivatives can induce genotoxic stress through DNA intercalation, strand cleavage, and reactive oxygen species (ROS) generation, thereby activating mitochondrial apoptosis pathways [52].

**Figure 12:**
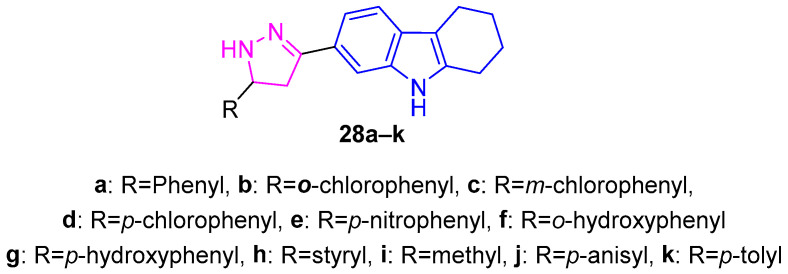
Pyrazoline-THCz hybrids as multifunctional antitumor agents.

Kamble et al. synthesized a series of pyrazoline-substituted THCz derivatives and evaluated their antitumor activities [52]. SAR revealed that electron-donating groups enhanced antitubercular effects (MIC < 5 μg/mL), while electron-withdrawing p-chloro and p-nitro derivatives (**28b**–**c**) induced complete DNA cleavage, suggesting enhanced DNA interaction. However, antitumor activity of these compounds was modest; only **28d**–**f** showed activity against A498 and A549 cells, correlating with hydrophobicity and hydrogen-bond donor capacity (Figure 12). Although not directly targeting specific proteins, these hybrids induce DNA damage via intercalation and radical-mediated cleavage, offering structural insights for developing DNA-targeted antitumor leads.

**Figure 13:**
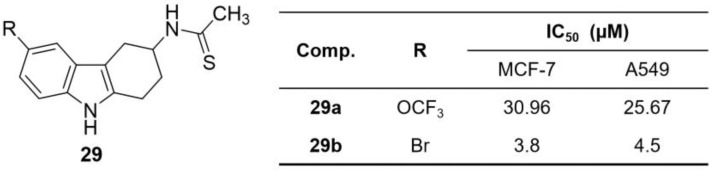
Thioamide-substituted THCz derivatives as multi-target antitumor agents.

Makowiec et al. synthesized a novel series of amide- and thioamide-substituted THCz derivatives **29**, incorporating various aromatic heterocycles at the 3-position via amide linkages [53]. Thioamide derivative **29b** emerged as a potent inhibitor with IC_50_ values of 3.8 μM and 4.5 μM against MCF-7 and A549, respectively (Figure 13). SAR analysis elucidated that the thioamide group was a key determinant of potency. Substituents at the 6-position were also crucial; compounds bearing strong electron-withdrawing groups such as trifluoromethoxy (**29a**) or bromine (**29b**) exhibited the greatest potency. Moreover, a synergistic effect was observed between the 3-position amide/thioamide and 6-position electron-withdrawing substituents. Notably, compound **29b** exhibited anti-angiogenic and tumor-growth inhibitory activity in vivo, suggesting therapeutic potential. While the precise molecular targets require elucidation, these findings provide a strong foundation for developing novel antitumor leads.

**Figure 14:**
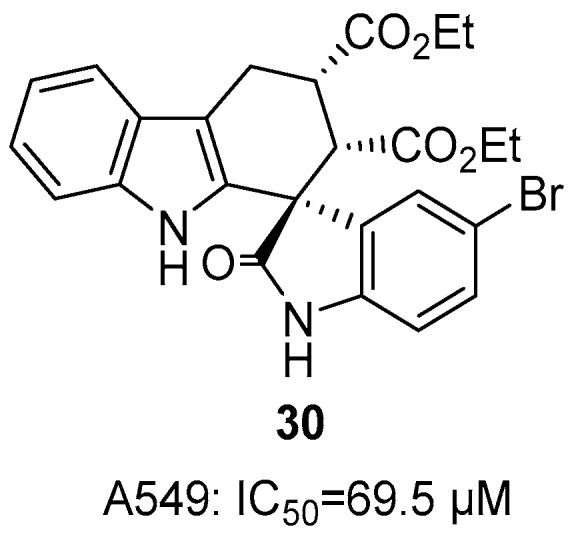
Spirooxindole-THCz derivatives as anti-tumor activities.

Erande et al. developed a series of spirooxindole-THCz (SOTC) derivatives and evaluated their antitumor activities [54]. Compound **30** exhibited moderate inhibitory activity against A549 and SKOV3 (IC_50_ ≈ 69.5 μM), with mechanistic studies revealing induction of ROS-mediated mitochondrial dysfunction and apoptosis (Figure 14). Complementary in silico docking against key oncology targets (e.g., EGFR, PI3K, MUC16) demonstrated strong binding affinities, highlighting the potential of these scaffolds as modulators of cancer-relevant pathways.

**Figure 15:**
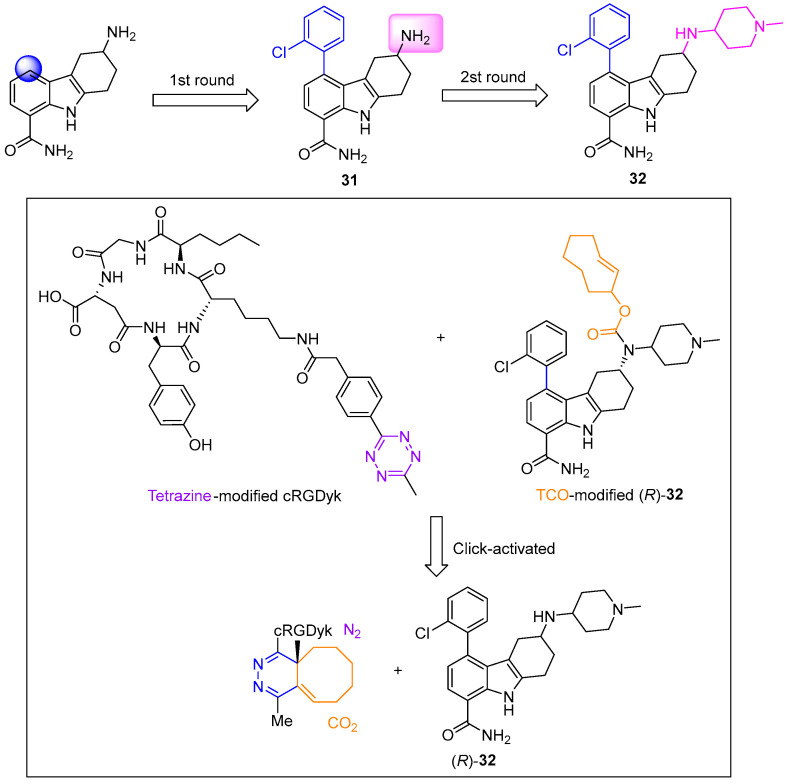
Discovery and characterization of potent antitumor THCz derivatives via a deep learning-driven cascade model.

Wang et al. employed a deep learning cascade model (XGBoost classifier + BART generative model) to discover THCz derivatives with antitumor activities [55]. Two potent THCz derivatives, **31** and **32** were discovered, exhibiting strong antiproliferative effects across multiple cancer cell lines—including drug-resistant variants—with IC_50_ values ranging from 0.2 to 45 nM. Further investigation revealed that the (*R*)-enantiomer of **32** upregulates p53 expression and induces mitochondria-dependent endogenous apoptosis. In vivo studies demonstrated significant tumor growth inhibition in xenograft models, while a click-activated prodrug strategy ((*R*)-**32**-TCO combined with cRGDyk-Tz) enabled targeted drug activation at tumor sites, minimizing systemic toxicity (Figure 15). This work highlights the efficacy of deep learning in accelerating phenotypic drug discovery and introduces a promising new chemotype for cancer therapy coupled with an innovative prodrug delivery system for enhanced selectivity and safety.

#### 2.4.3. Targeting Epigenetic Regulation and Transcriptional Stress Defense

Histone deacetylases (HDACs) [56,57], the transcription factor Nrf2 [58], and Hippo pathway effectors YAP/TAZ [59] are critical nodes through which tumor cells maintain proliferation, resist stress, and evade chemotherapy. THCz derivatives have demonstrated significant potential in targeting these epigenetic and transcriptional regulatory programs.

**Figure 16:**
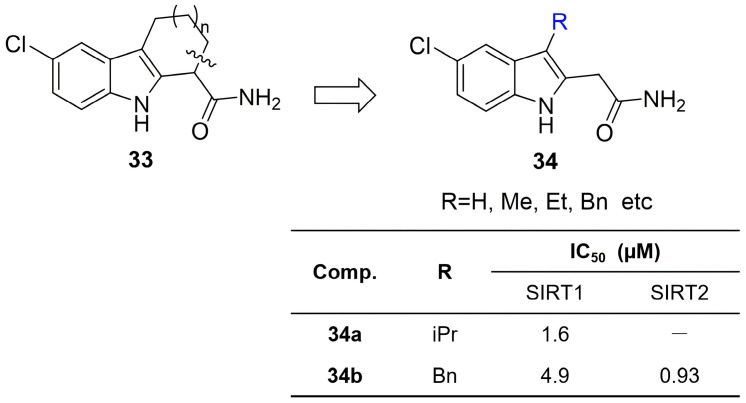
Development of novel indole-based SIRT1/SIRT2 inhibitors derived from THCz.

Hanna et al. designed and synthesized a series of achiral indole derivatives based on the known SIRT1-selective inhibitor **33** [60]. SAR analysis revealed that unsubstituted or small methyl groups at the indole 3-position showed negligible activity; extending the alkyl chain to ethyl or isopropyl progressively enhanced SIRT1 inhibition. The isopropyl-substituted derivative **34a** exhibited the most potent SIRT1 inhibition (IC_50_ = 1.6 μM) and improved selectivity over the lead compound **33**. In contrast, the benzyl-substituted analog **34b** displayed potent dual inhibition of SIRT1 and SIRT2 (IC_50_ = 4.9 μM and 0.93 μM, respectively) alongside broad-spectrum cytotoxicity (Figure 16). Mechanistically, this class of inhibitors functions by blocking SIRT1-mediated deacetylation of p53, thereby elevating acetylated p53 levels and activating p53-dependent apoptotic pathways. This study not only confirmed that eliminating the chiral center preserves bioactivity but also successfully identified both a highly selective SIRT1 inhibitor and a promising dual-target inhibitor with potent cytotoxicity.

**Figure 17:**
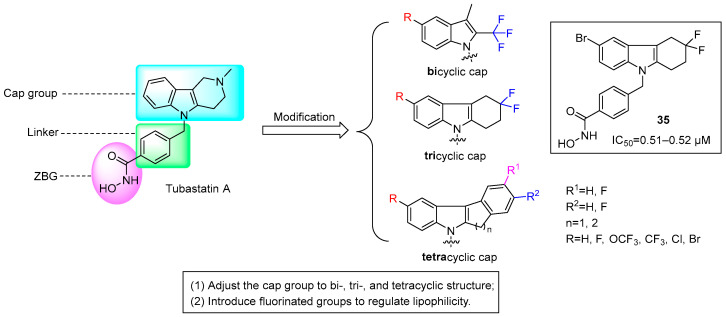
THCz compound as antitumor agents targeting HDAC6.

Zeng et al. synthesized 16 fluorinated-THCz derivatives based on Tubastatin A [58]. By incorporating bicyclic, tricyclic, and tetracyclic “cap groups” along with strategic fluorine substitution, they markedly enhanced HDAC-targeted inhibitory activity and broadened the antitumor spectrum. SAR analysis revealed a distinct effect of cap group complexity: tricyclic derivatives consistently exhibited the strongest and broadest in vitro antitumor activity, outperforming both bicyclic and tetracyclic analogs. Fluorination significantly enhanced binding affinity within the catalytic pocket of HDAC6 and improved cell membrane permeability. Compound **35** displayed potent activity against both SUNE1 nasopharyngeal carcinoma and MDA-MB-231 breast cancer cells, with IC_50_ values of 0.51–0.52 μM (Figure 17). Molecular docking confirmed that these derivatives bind stably to the HDAC6 active site, with low binding energies ranging from −6.54 to −9.84 kcal/mol. Key interactions include zinc ion chelation, hydrogen-bond networks, π-π stacking, and π-cation contacts.

**Figure 18:**
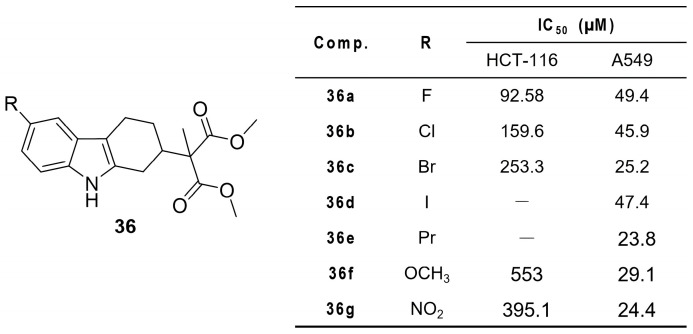
THCz compounds as Nrf2 modulators for inhibiting cancer cell growth.

Madhunapantula et al. designed THCz-based Nrf2 modulators (**36**) that stabilize the Nrf2-Keap1 interaction, preventing Nrf2 nuclear translocation and suppressing antioxidant gene transcription, thereby inducing cell cycle arrest and apoptosis [61]. SAR revealed a clear halogen preference at C-6: F > Cl ≈ Br > I/alkyl. The fluoro analog **36b** exhibited the strongest activity in Nrf2-overexpressing A549 cells (IC_50_ = 45.9 μM), suggesting that higher electronegativity enhances binding to Keap1 residues such as arginine (Figure 18). These compounds showed selective cytotoxicity toward high-Nrf2 tumors, confirming on-target activity via the Nrf2 pathway.

**Figure 19:**
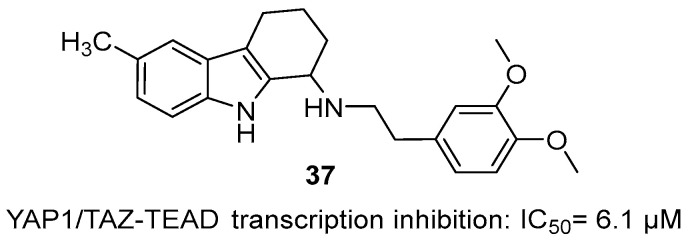
THCz compound inhibits bladder cancer progression by suppressing YAP1/TAZ.

Suzuki et al. identified a novel THCz compound **37** that inhibits bladder cancer progression [62]. This compound activates LATS1, promoting YAP1/TAZ phosphorylation, cytoplasmic retention, and proteasomal degradation. This inhibits YAP1/TAZ-TEAD transcription, suppressing proliferation and inducing apoptosis. In a nude mouse xenograft model, treatment with **37** led to significant tumor growth inhibition, with particularly promising efficacy against refractory basal and double-negative subtypes of bladder cancer (Figure 19). The critical dependence of this mechanism on YAP1/TAZ was confirmed by the complete abrogation of antitumor activity in YAP1/TAZ double-knockout cells, underscoring its on-target action. This research offers valuable mechanistic insights and a promising lead structure for the development of multi-targeted, THCz-based antitumor agents.

#### 2.4.4. Targeting Signal Transduction Pathways

Targeting receptor tyrosine kinases (VEGFR-2, EGFR, ErbB-2) and their downstream effectors (ERK, Rb) is a cornerstone of cancer therapy [63,64]. Through multi-target hybrid design or structural optimization, THCz derivatives have achieved synergistic intervention of multiple signaling pathways.

Abdu-Allah et al. hybridized THCz with 5-arylidene-4-thiazolidinone to target VEGFR-2, EGFR, and tubulin [13,65,66,67]. Among the synthesized compounds, 6-Chloro derivatives **38a** and **38b** showed potent activity against leukemia/lymphoma cells (IC_50_ as low as 4.53 μM). **38a** exhibited exceptional VEGFR-2 inhibition (IC_50_ = 0.26 μM), 1.5-fold more potent than sorafenib. SAR analysis indicated that the substituents on the phenyl ring significantly influenced biological activity: hydroxy groups at the ortho- or para-positions (as in **38a** and **38b**) enhanced nonproliferation effects in leukemia/lymphoma cells and improved VEGFR-2 inhibition; a para-methoxy substituent (as in **38c**) maintained strong VEGFR-2 inhibitory potency but markedly reduced activity in colon cancer cell lines. Moreover, the introduction of chlorine at the 6-position generally enhanced bioactivity, likely by optimizing electronic distribution or facilitating halogen bonding with target proteins. Molecular docking confirmed that active compounds bind stably within the active sites of VEGFR-2, EGFR, and tubulin.

**Figure 20:**
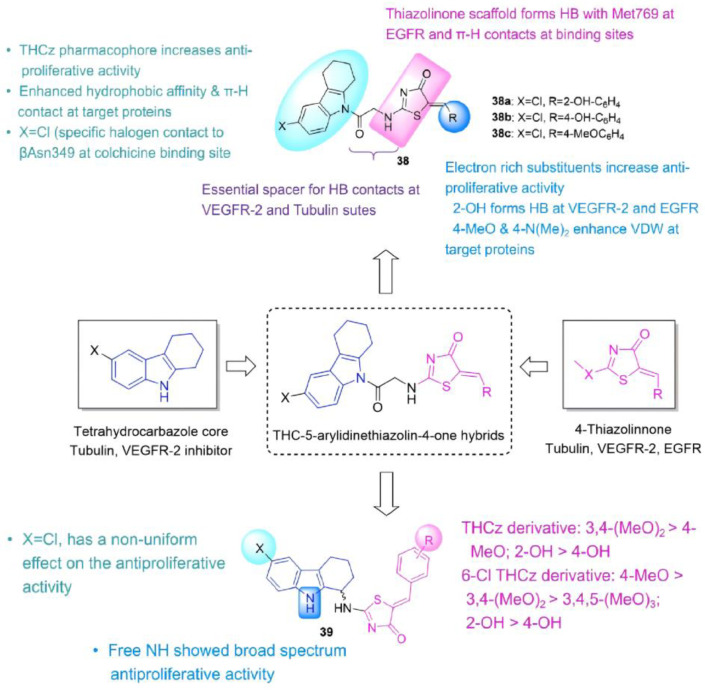

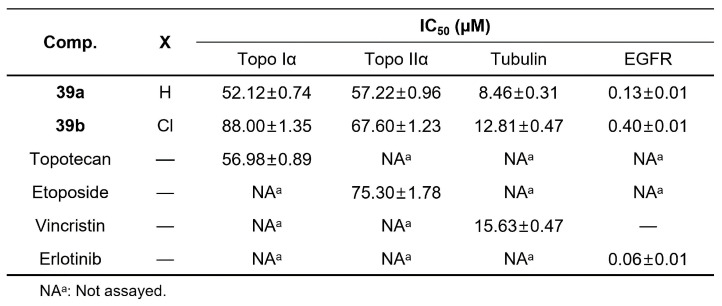
Design and SAR analysis of THCz and 5-arylidene-4-thiazolidinone hybrids as potential antitumor agents.

Subsequent optimization relocated thiazolidinone to C-1, shifting conformation from T-shaped to bent-linear for improved target fit, preserving topoisomerase binding and strengthening hydrogen-bond interactions with tubulin and EGFR [68]. Among these new hybrids, compounds **39a** and **39b** exhibited the most potent antiproliferative activity against Jurkat, U937, and HCT-116 cancer cell lines (IC_50_ = 1.44–6.75 μM) with low cytotoxicity toward normal HME1 cells (IC_50_ > 50 μM), demonstrating broader and stronger efficacy than earlier analogs. **39a** simultaneously inhibited Topo Iα (IC_50_ = 52.12 μM), Topo IIα (57.22 μM), EGFR (0.13 μM), and tubulin polymerization (8.46 μM), performing comparably or superior to standard inhibitors. It also induced G0/G1 cell cycle arrest and promoted apoptosis in Jurkat cells. Molecular dynamics simulations revealed that the (*S*)-enantiomer of **39a** binds more stably to Topo I/II via THC-core intercalation into DNA, while the (*R*)-enantiomer preferentially engages EGFR through halogen bonding and hydrophobic interactions. In tubulin, the (*S*)-enantiomer forms hydrogen bonds and π-stacking with residues such as Asn258 and Lys352, whereas the (*R*)-enantiomer occupies the EGFR hydrophobic pocket similarly to erlotinib (Figure 20). The complementary binding profiles across targets suggest the racemic mixture may offer synergistic multitarget advantages over single enantiomers.

**Figure 21:**
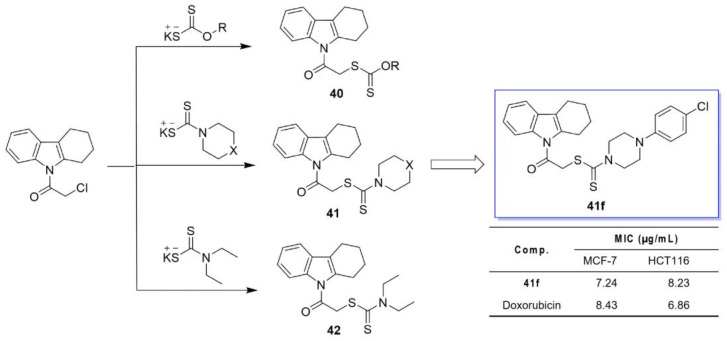
THCz-dithioate hybrid molecules with antiproliferative activity.

El-Nassan et al. designed three classes of THCz-dithioate hybrids: O-alkyl dithiocarbonates (**40**), N,N-disubstituted dithiocarbamates (**41**), and dialkyl dithiocarbamates (**42**) [69]. SAR analysis indicated that antitumor activity strongly depended on the structural characteristics of the substituents attached to the dithioate moiety. Derivatives containing a piperazine ring (**41**) demonstrated the most potent inhibitory activity. Notably, the 4-chlorophenylpiperazine-substituted derivative **41f** exhibited the strongest activity, with an IC_50_ of 7.24 nM against MCF-7 cells, outperforming the positive control doxorubicin (Figure 21). Sensitivity to different structural classes varied across cancer cell lines: MCF-7 cells showed greater sensitivity to alkyl dithiocarbonates, while HCT116 cells responded more strongly to heterocyclic dithiocarbonates. Although direct target validation was not conducted, the authors proposed that the antitumor effects likely involve multiple mechanisms, including inhibition of EGFR and ErbB-2 kinase, suppression of carbonic anhydrase activity, and decomposition to release carbon disulfide, ultimately contributing to oxidative stress and promoting apoptosis.

**Figure 22:**
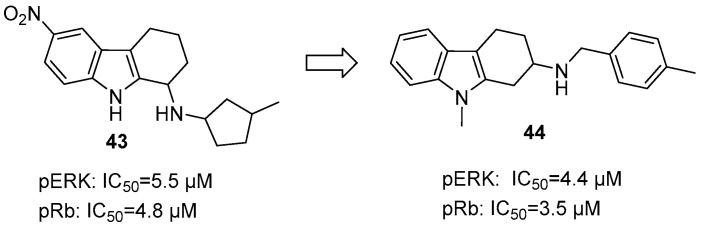
1-amino-THCz compounds as dual inhibitors of pERK and pRb.

Pandit et al. identified 1-amino-THCz compound **43** as a dual inhibitor of pERK and pRb (IC_50_ = 5.5 μM and 4.8 μM, respectively) [70]. Expanding on this lead, a series of 2-amino-THCz derivatives was developed. Among them, compound **44** emerged as the most potent, establishing the first small molecule capable of concurrently targeting both pERK and pRb, with IC_50_ values of 4.4 μM and 3.5 μM, respectively (Figure 22). SAR studies revealed that repositioning the amino group from C-1 to C-2 and incorporating aromatic substituents such as benzyl or p-tolyl substantially improved activity. Further potency gains were achieved through methyl substitution at the C-6 position. N-methylation (as in compound **44**) had a modest effect, indicating that this site is amenable to further structural optimization. Mechanistically, this compound suppresses ERK phosphorylation (disrupting MAPK signaling) and inhibits Rb phosphorylation, preventing CDK/cyclin-mediated inactivation and inducing G_1_-S arrest.

#### 2.4.5. Targeting the Tumor Immune Microenvironment

Tumor-associated macrophages (TAMs) exhibit functional plasticity between antitumor (M1) and protumor (M2) phenotypes. During tumor progression, TAMs typically polarize toward the M2 state, promoting immunosuppression, angiogenesis, and metastasis. Reprogramming TAMs from M2 to M1 represents a promising immunotherapeutic strategy to restore antitumor immunity [71].

**Figure 23:**
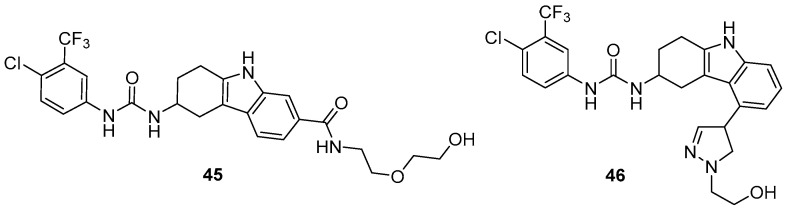
Ureido THCz derivatives for cancer treatments through targeting tumor-associated Macrophages.

Chen et al. identified urea-THCz **45** from phenotypic screening as an inducer of TNF-α, an M1 marker [72]. Optimization yielded **46**, which dose-dependently upregulated M1 markers (IL-1β, TNF-α, iNOS, IL-12α) in RAW 264.7 cells and reduced IL-4-induced M2 polarization. In a Lewis lung carcinoma model, **46** (5 mg/kg) significantly inhibited tumor growth and synergized with anti-PD-1 therapy. Mechanistically, **46** promotes TNF-α expression and modulates STAT6 signaling, driving TAM repolarization toward an M1-like phenotype (Figure 23).

#### 2.4.6. Targeting Mitochondrial TSPO for Photodynamic Therapy

TSPO, an 18 kDa outer mitochondrial membrane protein, is overexpressed in aggressive cancers (glioblastoma, breast, ovarian, colorectal). Its upregulation correlates with tumor progression and poor prognosis, making it a valuable biomarker and therapeutic target [73].

**Figure 24:**
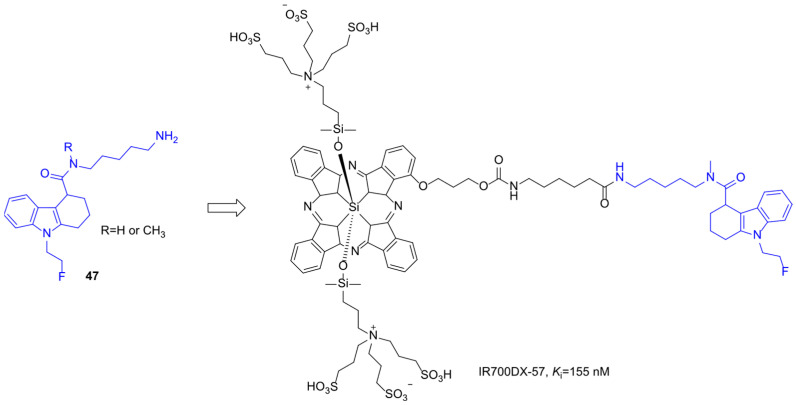
Translocator protein (TSPO)-targeted agents for photodynamic therapy of cancer.

Bai et al. conjugated THCz (**47**) and quinazoline-based TSPO ligands to IR700DX to create targeted photosensitizers (TSPO-PS) [74]. SAR analysis showed that N-methylation of these ligands substantially increased TSPO binding affinity, shifting K*ᵢ* values from the micromolar to the nanomolar range, likely due to optimized interactions within the TSPO binding pocket (Figure 24). Upon irradiation, TSPO-PS generated ROS, inducing mitochondrial apoptosis. Some lysosomal uptake contributed to phototoxicity. These findings highlight the importance of balancing target specificity with cellular internalization in designing TSPO-directed photosensitizers, while also demonstrating the broader potential of THCz scaffolds in precision drug design.

#### 2.4.7. Other Structural Modifications and Studies with Undefined Targets

**Figure 25:**
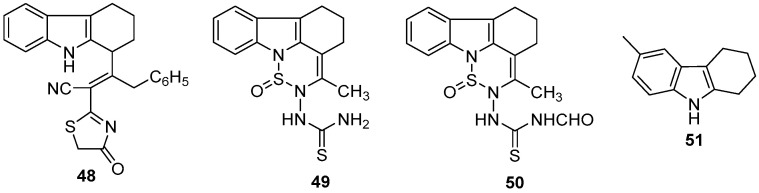
THCz derivatives as antitumor activity with undefined targets.

Several THCz derivatives have shown significant antitumor activity in screening assays, although their precise molecular targets remain to be elucidated or are in early stages of investigation (Figure 25).

Ismail et al. designed and synthesized a series of 1-substituted and 1,9-disubstituted THCz derivatives, incorporating various heterocycles (thiazole, thiadiazine, pyrazole) and functional groups (acyl, cyano, amido) to enhance antitumor activity [75]. Among these derivatives, compound **48** demonstrated the most potent activity, with an ED_50_ of 26 μg/mL. Additionally, sulfur-containing heterocyclic derivatives (compounds **49** and **50**) exhibited moderate to strong activity, indicating that electron-withdrawing groups and heterocyclic structures positively contribute to enhancing potency.

Mahadevan et al. reported the 6-methyl-substituted THCz derivative **51** showed highly selective and potent inhibitory activity against the lung cancer cell line Calu-1, with an IC_50_ of 2.5 nM, while exhibiting low toxicity in normal cells, indicating promising potential for further development [76]. However, the specific molecular targets of these compounds were not investigated in detail in this study.

#### 2.4.8. Cross-Target SAR for Antitumor THCz Derivatives

As summarized in Table 4, despite the diversity of antitumor mechanisms targeted by THCz derivatives—ranging from microtubule disruption and epigenetic modulation to multi-kinase inhibition and oxidative stress induction—common design principles have emerged. Universally, C-6 halogenation (particularly Br, Cl, or F) enhances potency across multiple targets; stereochemistry at C-1 requires careful control, often dictating target selectivity between enantiomers; N-9 modifications (methyl, acyl, or bulky caps) critically modulate target engagement and metabolic stability; and linker optimization in hybrid designs is essential for achieving multi-target synergy. These cross-target insights provide a practical framework for the rational design of next-generation THCz-based antitumor agents.

### 2.5. Agents for Autoimmune Diseases

Bruton’s tyrosine kinase (BTK) is a key regulator of B-cell receptor (BCR) signaling and other immune pathways, controlling B-cell development, activation, and proliferation [77]. Dysregulated BTK activity is closely linked to autoimmune diseases such as rheumatoid arthritis and systemic lupus erythematosus. Inhibiting BTK can therefore suppress aberrant BCR signaling and excessive B-cell activation, offering a promising therapeutic strategy for these conditions [78,79].

**Figure 26:**
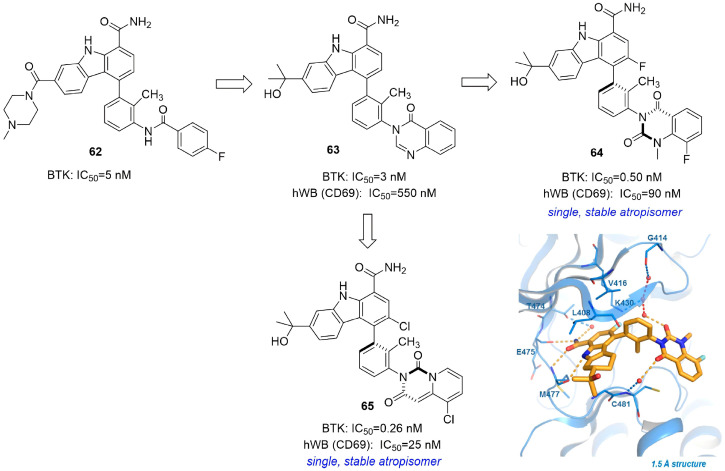
Development of the THCz-based BTK inhibitors for autoimmune diseases.

Tino et al. optimized carbazole lead **52** to improve selectivity and pharmacokinetics [80]. Replacing a labile benzamide with a cyclic lactam and modifying piperazine to a tertiary alcohol yielded quinazolinone **53** (BTK IC_50_ = 2.8 nM; 84–100% oral bioavailability; efficacy in arthritis models), leading to its selection as a clinical candidate. However, **53** suffered from four interconverting atropisomers, causing weak human whole-blood activity (IC_50_ = 550 nM). Conformational locking via a second carbonyl and C-3 fluoro substitution produced stable single isomer **54** (BTK IC_50_ = 0.50 nM; whole-blood IC_50_ = 90 nM; >10,000-fold selectivity over JAK2), which advanced to Phase II trials for rheumatoid arthritis [81]. Further optimization using a pyridopyrimidinedione core with halogen-locked atropisomers yielded **55** (BTK IC_50_ = 0.26 nM; whole-blood IC_50_ = 25 nM; 3800-fold selectivity over JAK2) with improved efficacy and safety [82] (Figure 26). This work validated conformational constraint as a key strategy for optimizing BTK inhibitors.

**Figure 27:**
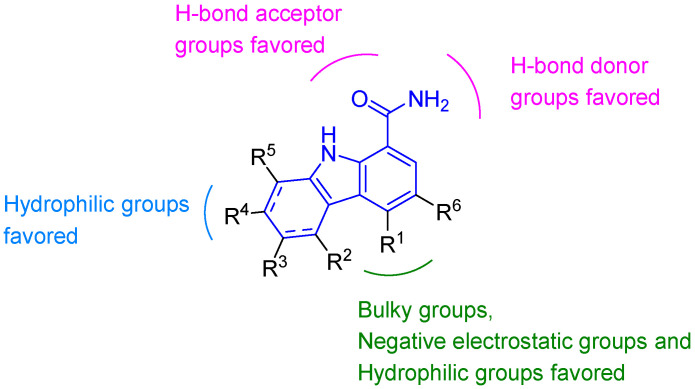
SAR analysis of the THCz-based BTK inhibitors.

Du et al. developed 3D-QSAR models for 132 analogs of **54** to define BTK inhibition requirements [83]. Molecular docking revealed the carbazole core binds ATP-site via hydrogen bonds (Gly475, Met477) and π-π stacking (Tyr466). Key interactions included hydrophobic contact between the R^6^ substituent (e.g., Cl) and Glu407/Asp539; projection of the R^1^ phenyl ring into a “floor loop” region (Asn484, Leu483, Arg525); and a hydrogen bond from the R^4^ hydroxyl to Ala478. These models revealed key design principles: bulky, electronegative substituents at the para- or ortho-position of R^1^ enhance activity; the N-H at position 1 of carbazole is an essential hydrogen-bond donor; a hydrophilic group at R^4^ stabilizes binding via Ala478; and bulky R^3^ groups should be avoided to prevent steric hindrance. The carbazole or saturated THCz core offers a rigid hydrophobic scaffold, with saturation further improving solubility and metabolic stability (Figure 27). Together, these insights provide a clear theoretical framework for designing potent and selective BTK inhibitors.

**Figure 28:**
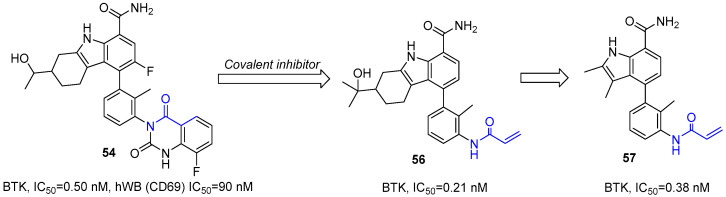
THCz covalent inhibitors targeting BTK.

Based on co-crystal structures showing proximity of the quinazolinone of **54** to Cys481, Tino et al. replaced it with electrophilic warheads, such as acrylamide or vinyl sulfone, leading to the successful development of irreversible inhibitors with significantly enhanced potency, exemplified by compound **56** (BTK IC_50_ = 0.21 nM) [84]. Further optimization “opened” the THCz core to 2,3-dimethylindole-7-carboxamides. This series maintained excellent activity (e.g., compound **57**, BTK IC_50_ = 0.38 nM) while significantly lowering molecular weight, heavy-atom count, and LogP, thereby improving ligand efficiency and drug-likeness (Figure 28). Although the resulting indole-based covalent inhibitors still face challenges in in vitro metabolic stability, their potent inhibition, high selectivity, and optimized physicochemical properties offer a promising new scaffold for developing low-dose, highly effective, and safe covalent BTK inhibitors. This series of studies clearly illustrates a rational evolution from complex natural structures to simplified privileged scaffolds, serving as a successful case study in structure-based drug design.

### 2.6. Antidiabetic Agents

Type 2 Diabetes Mellitus (T2DM) poses a major global health challenge due to its high prevalence and complications [83]. AMP-activated protein kinase (AMPK), a master regulator of cellular metabolism, has emerged as a compelling therapeutic target. Its activation enhances glucose uptake and fatty acid oxidation while suppressing gluconeogenesis and lipogenesis, addressing multiple facets of T2DM pathophysiology [85].

Tang et al. employed a scaffold-hopping strategy to design novel THCz derivatives as potent AMPK activators [14]. Lead compound **58** emerged as a candidate, demonstrating glucose uptake 82.5% above baseline in HepG2 cells—surpassing metformin (63.7%). In db/db diabetic mice, **58** effectively lowered blood glucose and triglycerides comparable to pioglitazone without inducing weight gain. Molecular docking revealed that the active enantiomer, (*R*)-**58**, forms multiple hydrogen bonds and non-bonded interactions with the α and β subunits of AMPK. In contrast, (*S*)-**68** lacked key hydrogen bonds and exhibited reduced activity, demonstrating stereospecific binding. SAR analysis indicated that electron-withdrawing groups on the C-6 benzyloxy ring and N-9 position enhanced potency, whereas electron-donating or bulky substituents diminished activity. Cyclization between C-6 and C-7 generally reduced efficacy, and chirality at the stereocenter was critical, with the levorotatory isomer consistently exhibiting superior activity. In an HFD/STZ diabetic mouse model, **58** robustly improved glucose metabolism and insulin sensitivity.

**Figure 29:**
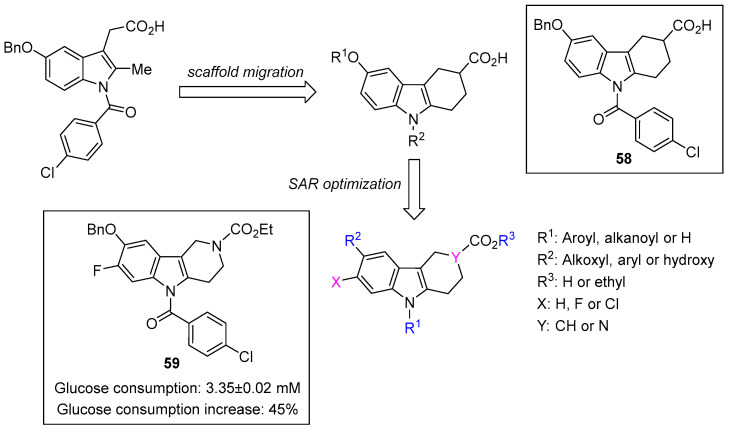
THCz derivatives as potent and safe AMPK activators for T2DM.

Building on these insights, Tang’s group designed an expanded series of THCz analogs, with compound **59** emerging as the most potent [15]. Western blot analysis confirmed that **59** strongly promotes AMPKα phosphorylation at Thr172, consistent with the mechanism of **58**. Molecular dynamics simulations indicated that **59** exhibits a more favorable binding of free energy with AMPK α1β1γ1 (−28.27 kcal/mol) than **58** (−24.64 kcal/mol), due in part to a key hydrogen bond formed by the C-7 fluorine with Asn48. SAR analysis revealed that substitution at N-1 with simple acyl groups (e.g., isobutyryl) moderately retained activity; fluorine substitution at C-7 in aza-THCz derivatives was optimal, while chlorine was preferred in the classic THCz scaffold. Notably, the aza-THCz scaffold eliminated chirality constraints and boosted potency. Compound **59** increased glucose consumption by 45%, exceeding both **58** (38.6%) and metformin (37.6%). The C-6 benzyloxy group proved critical for activity (Figure 29).

This study successfully identified a class of aza-THCz derivatives represented by compound **59** as potent AMPK activators and promising candidates for the treatment of type II diabetes. Future research should focus on improving their oral bioavailability and in vivo efficacy and safety profile.

### 2.7. Agents for Central Nervous System (CNS) Diseases

**Figure 30:**
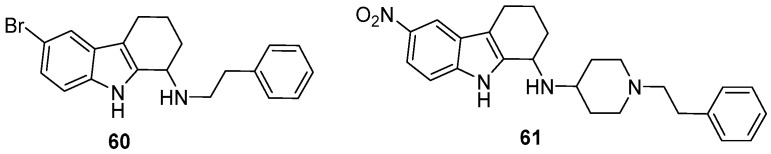
THCz compounds with restored disturbed Ca^2+^ homeostasis.

Huntington’s disease (HD) is driven by CAG expansion in the huntingtin (HTT) gene, leading to cellular dysfunction including disrupted store-operated calcium entry (SOCE) [86]. Czeredys et al. demonstrated that THCz derivatives **60** and **61** restore SOCE in medium spiny neurons from YAC128 HD mice, reducing pathological calcium influx by ~14% and ~10%, respectively, to near wild-type levels [17] (Figure 30). SAR analysis identified the THCz core, a 6-bromo substituent, and a phenethylamine side chain as critical for activity. These compounds do not affect store release but selectively inhibit Ca^2+^ influx and stabilize mitochondrial membrane potential.

**Figure 31:**
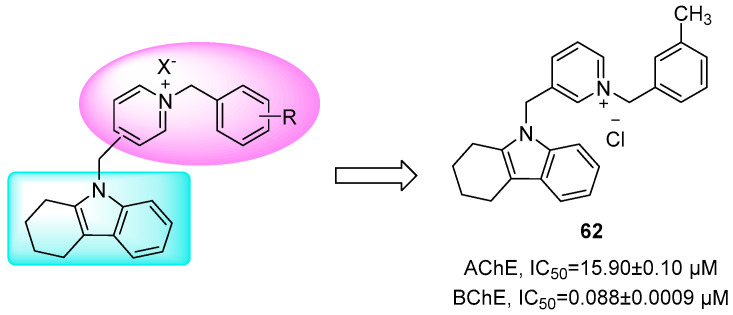
THCz-benzylpyridine hybrids as potent and selective BuChE inhibitors.

Alzheimer’s disease (AD) involves cognitive decline and reduced acetylcholine (ACh) levels. Butyrylcholinesterase (BuChE) has emerged as a promising therapeutic target due to its upregulation in AD and potential for fewer cholinergic side effects than AChE inhibition [87,88]. Amini et al. designed THCz-benzylpyridine hybrids as selective BuChE inhibitors [16]. Compound **62** demonstrated exceptional potency (IC_50_ = 0.088 μM against BuChE) and acted as a dual-site inhibitor, engaging both the catalytic and peripheral sites—an interaction enabled by optimal fit within BuChE’s larger acyl pocket. Beyond cholinesterase inhibition, **62** exhibited neuroprotection, β-secretase (BACE1) inhibition, and suppression of Aβ aggregation, supporting a multi-target anti-AD profile. SAR revealed that a 3-pyridylmethyl substituent and p-methyl group on the phenyl ring enhanced activity, while fluorine substitution was detrimental (Figure 31).

**Figure 32:**
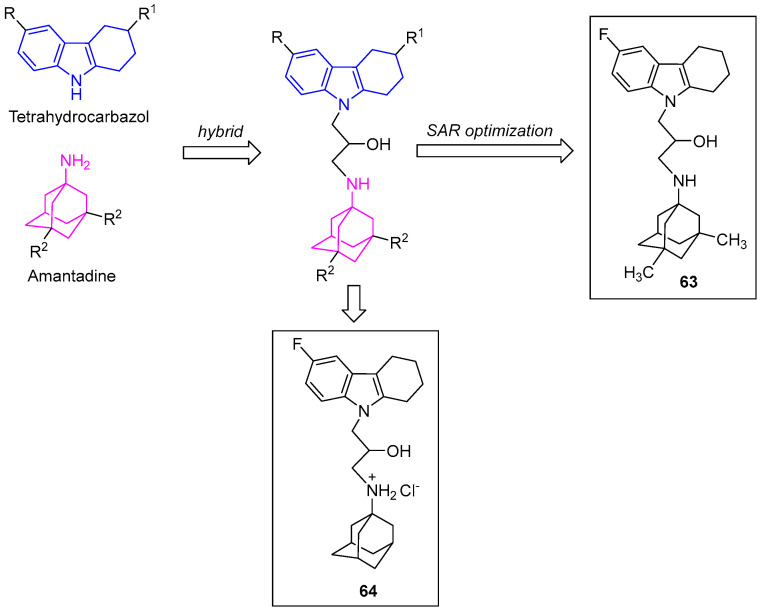
Aminoadamantane-THCz conjugate as a selective mitochondrial calcium uptake inhibitor.

In 2017, Aliev et al. designed amino-adamantane-THCz conjugate **63** linked via a 2-hydroxypropyl group. Lead compound **63** demonstrated potent and selective BuChE inhibition (IC_50_ = 5.43 µM) with minimal activity against AChE and carboxylesterase (IC_50_ > 20 µM). **63** also inhibits the mitochondrial calcium uniporter (MCU), preventing glutamate-induced Ca^2+^ overload and preserving membrane potential [89]. Abramov’s group reported structurally similar conjugate **64**. Despite sharing the same scaffold, **64** did not act on the canonical MCU pathway; instead, it selectively suppressed mitochondrial Ca^2+^ uptake induced by low Ca^2+^ concentrations, achieving neuroprotection without disrupting physiological Ca^2+^ signaling [90] (Figure 32). These compounds reveal the therapeutic potential of the THCz scaffold in precisely modulating pathological Ca^2+^ signaling through a dual-pharmacophore strategy.

**Figure 33:**
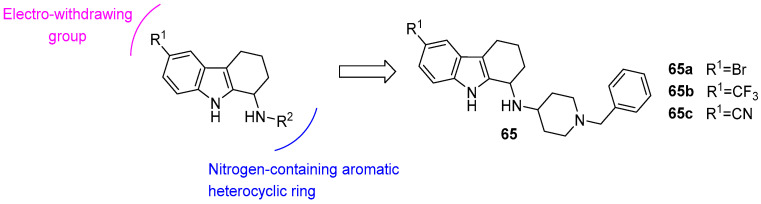
THCz compounds as novel multifactorial drug candidates for treatment of Alzheimer’s disease.

Intracellular calcium dysregulation is an early pathological feature in AD, closely linked to endoplasmic reticulum (ER) dysfunction. Stabilizing ER calcium homeostasis thus represents a critical therapeutic strategy. As reported by Herms et al., THCz derivatives **65a**–**c** effectively suppress excessive calcium release from the ER in cellular models carrying familial AD-associated mutations, thereby restoring normal calcium signaling. Beyond calcium modulation, these compounds also enhance mitochondrial membrane potential, improve mitochondrial function, and selectively inhibit BACE1 activity, reducing amyloid-β peptide production without affecting α- or γ-secretase function—a profile that minimizes interference with essential physiological pathways [91] (Figure 33).

**Figure 34:**
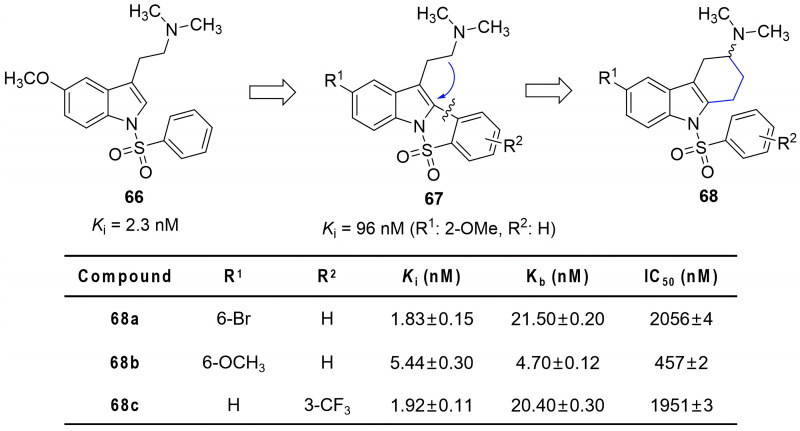
Design of the 5-HT_6_ selective inhibitor and the representative compounds.

In Alzheimer’s disease drug development, the serotonin 6 (5-HT_6_) receptor has emerged as a promising cognitive target due to its CNS enrichment [92]. Nirogi et al. designed novel THCz-based 5-HT_6_ antagonists by cyclizing the side-chain amine of tryptamine that leads to the C-2 indole position, forming a rigidified scaffold **68** that preserved key pharmacophoric elements—an arylsulfonyl hydrogen-bond acceptor and a cationic tertiary amine. SAR revealed that 6-bromo or 6-methoxy substitution significantly enhanced binding (e.g., **68a**, *K*_i_ = 1.83 nM; **68b**, *K*_i_ = 5.44 nM), with potency order Br > OCH_3_ > H > Cl > F. On the sulfonyl-linked B-ring, meta-trifluoromethyl substitution was particularly favorable (e.g., **68c**, *K*_i_ = 1.92 nM). The dimethylamino group proved essential for high affinity, supporting the cationic center pharmacophore model (Figure 34). Lead compound **68b** demonstrated potent antagonism of 5-HT-induced cAMP accumulation (IC_50_ = 457 nM), favorable oral bioavailability (26%), high brain penetration (brain/plasma ratio = 7.6), and robust pro-cognitive effects in a rat novel object recognition model. High selectivity over related targets (5-HT_1_A, 5-HT_2_A, D_2_, SERT) further supports its potential as a candidate for Alzheimer’s disease and related cognitive disorders.

These findings suggest that THCz-based compounds represent a promising multi-target therapeutic strategy for AD, capable of addressing several pathological processes simultaneously, and may be particularly suitable for early intervention in both familial and sporadic forms of the disease.

### 2.8. Agents with Other Bioactivities

**Figure 35:**
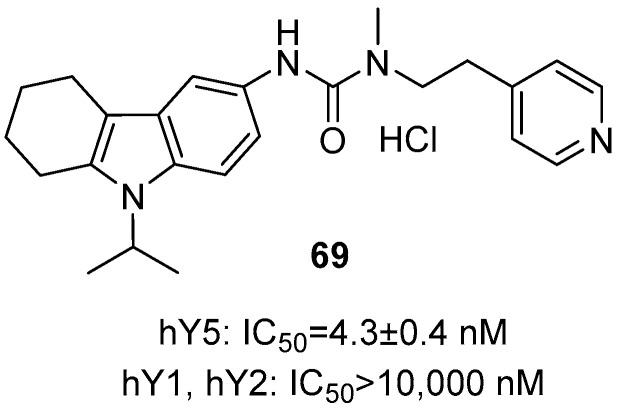
THCz compound as a novel and selective antagonist for neuropeptide Y5 receptor.

Neuropeptide Y (NPY), a widely distributed 36-amino acid peptide, is one of the most potent known orexigenic factors. Its effects are mediated through multiple receptor subtypes, with the Y5 receptor playing a key role in feeding regulation [93]. Kitaguchi and colleagues systematically evaluated THCz derivative as a Y5 receptor antagonist. Compound **69** demonstrated high affinity for Y5 (IC_50_ = 4.3 nM) and exceptional selectivity over Y1 and Y2 receptors (IC_50_ > 10,000 nM) (Figure 35). In vivo, **69** exhibited favorable oral bioavailability and blood–brain barrier penetration, selectively inhibiting feeding triggered by NPY, Y5-specific agonists, and fasting, without affecting responses to other orexigenic factors. Immunohistochemical analysis confirmed its central Y5-specific action, showing reduced c-Fos expression in Y5-positive neurons [94]. Given Y5’s potential roles in cognition and neuroprotection, compound **69** and its analogs also warrant further investigation for neurological disorders.

**Figure 36:**
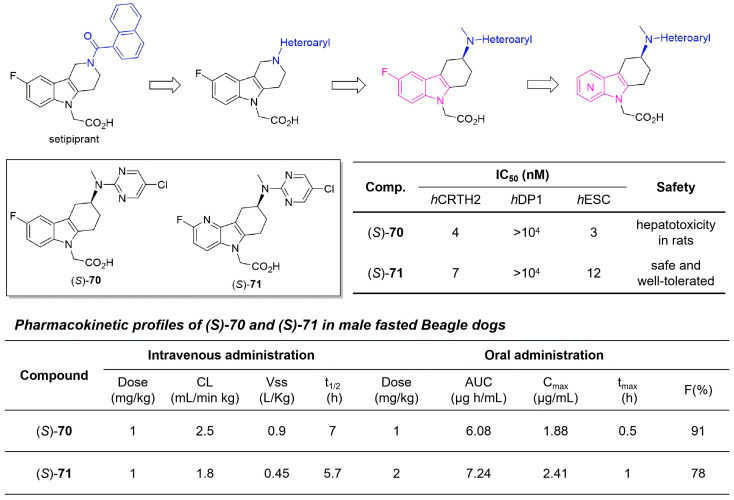
Development of CRTH2 antagonists and their activities and pharmacokinetic profiles.

The CRTH2 (DP2) receptor, selectively expressed on type 2 inflammatory cells such as Th2 cells, eosinophils, and basophils, mediates pro-inflammatory responses upon activation by prostaglandin D_2_ (PGD_2_), making it a promising therapeutic target for allergic diseases [95,96]. Although several antagonists have entered clinical trials, many have faced efficacy challenges, underscoring the need for improved agents. In 2023, Boss and colleagues developed an enhanced CRTH2 antagonist building on the clinical candidate setipiprant, aiming to overcome its plasma-driven potency loss while maintaining favorable pharmacokinetics and safety [97]. Their multi-stage optimization strategy began with replacement of the amide linker with aromatic heterocycles, yielding modest improvements. A more significant advance came from a scaffold hop to aminomethyl-THCz derivatives, which greatly enhanced potency and introduced chirality; the (*S*)-enantiomer **70** exhibited strong antagonism and good oral bioavailability but was halted due to rat hepatotoxicity. To address this toxicity, the indole core was replaced with an azaindole system, leading to (*S*)-**71**, a 5-fluoro-4-azaindole derivative that retained nanomolar potency, selectivity, and excellent pharmacokinetics while showing no hepatotoxicity in vitro (Figure 36). This work successfully obtained a potent, selective, and safer preclinical candidate for CRTH2-mediated allergic diseases.

The extensive biological activities and SAR insights discussed above are exemplified by a diverse array of THCz-based compounds that have advanced from initial hits to leads and, in some cases, clinical candidates. Table 5 consolidates the most representative compounds across all therapeutic areas, summarizing their core scaffolds, primary targets, key activity data, and current development stage. This compilation serves as a rapid-reference portfolio that illustrates how strategic modifications at privileged positions have been successfully translated into potent modulators against phylogenetically unrelated targets.

## 3. Strategies and Future Directions in THCz-Based Drug Design

To advance the field from opportunistic discovery toward rational engineering, we critically distill four emerging design paradigms that transcend individual targets, analyzing their mechanistic logic, structural implementation, and transitional bottlenecks. By decoding cross-target SAR patterns and deconstructing successful optimization campaigns, we aim to provide a practical framework for the development of next-generation THCz therapeutics.

### 3.1. Pharmacophore Hybridization

The THCz scaffold possesses a unique combination of moderate hydrophobicity, planar rigidity, and multiple modifiable vectors, making it an exceptional platform for pharmacophore hybridization. Rather than serving merely as a lipophilic anchor, the THCz core actively contributes to target engagement while enabling the precise spatial presentation of a second pharmacophore. This strategy has been exploited across multiple therapeutic domains with convergent design logic.

The fusion of THCz with a 2,4-diaminopyrimidine moiety (**4**) produced a first-in-class dual inhibitor simultaneously targeting bacterial SC and DHFR. Critically, systematic linker optimization established that a three-carbon methylene tether is optimal, balancing conformational freedom for simultaneous bivalent engagement without entropic penalty [22]. A parallel antitumor campaign merged THCz with 5-arylidene-4-thiazolidinone at either C-6 (**38**) or C-1 (**39**) and generated triple inhibitors of VEGFR-2, EGFR, and tubulin, with the point of attachment profoundly influencing target preference: C-6 conjugates favored kinase inhibition, whereas C-1 conjugates preferentially engaged topoisomerases [67,68]. This positional effect—hitherto unrecognized—suggests that the THCz core orients the appended pharmacophore into distinct regions of the proteomic landscape, offering a tunable vector for poly-pharmacology.

In neuroprotection, the conjugation of THCz with aminoadamantane via a 2-hydroxypropyl linker (**63**, **64**) yielded dual BuChE inhibitors and mitochondrial calcium uniporter (MCU) modulators [89,90]. The hydroxyl group on the linker is not merely a spacer but an active participant, forming critical hydrogen bonds that enhance both affinity and selectivity. This illustrates a broader principle: in hybrid design, the linker is not passive—it is a third pharmacophore.

Despite these successes, hybrid molecules frequently exceed favorable lipophilicity and molecular weight ranges, risking poor oral bioavailability and off-target promiscuity. The field lacks systematic comparative studies evaluating whether covalently linked hybrids outperform physical mixtures of individual pharmacophores. Future efforts should prioritize linker bioisosterism (e.g., triazole, oxadiazole) to reduce rotatable bonds and lipophilicity and employ proteomic profiling to deconvolute the true mechanism of synergistic hybrids.

### 3.2. Conformational Constraint

The saturated ring of THCz introduces a stereochemical handle absent in fully aromatic carbazoles, offering unique opportunities for conformational control. Successful campaigns across multiple targets demonstrate that strategic rigidification can simultaneously enhance potency, selectivity, and metabolic stability—provided the constraint is precisely matched to the target’s three-dimensional architecture.

The evolution of BTK inhibitors exemplifies this paradigm with exceptional clarity. Early lead **52**, despite nanomolar biochemical potency, suffered from atropisomerism and poor whole-blood activity. Stepwise conformational locking—first via quinazolinone formation (**53**), then through dual atropisomeric control with C-3 substitution (**54**, **55**)—progressively restricted rotational freedom, converting an entropically handicapped mixture into a single, bioactive conformer. This yielded a clinical candidate with >10,000-fold selectivity over JAK2 and 84–100% oral bioavailability. Crucially, the optimal locking group (F > Cl > Me > CN) followed a clear steric hierarchy, providing a generalizable guideline for atropoisomeric control in biaryl systems [80,81,82].

A parallel strategy emerged in 5-HT_6_ antagonist development. Cyclizing the flexible tryptamine side chain of **67** to form the tetracyclic THCz **68** locked the cationic amine and aryl sulfonyl pharmacophores into a fixed spatial relationship. This conformational preorganization improved receptor affinity by an order of magnitude and abolished off-target activity at 5-HT_1_A, D_2_, and SERT [92]. Notably, the cyclohexane ring in THCz is not merely a passive scaffold—its chair conformation projects the C-1 substituent into a specific vector, explaining the absolute stereochemical requirement observed in HPV inhibitors (**21**–**23**) [46,47], AMPK activators (**58**) [16], and CRTH2 antagonists (**70**–**71**) [97]. In each case, the (*R*)-enantiomer consistently outperformed its (*S*)-counterpart, a remarkable cross-target stereochemical convergence that implicates a conserved binding mode: the C-1 substituent likely occupies a hydrophobic pocket with stringent chiral discrimination.

While conformational constraint reliably improves affinity, its impact on selectivity is less predictable. In the BTK series, rigidification unexpectedly expanded selectivity margins; in other systems, it may collapse conformational ensembles essential for target discrimination. Moreover, atropoisomeric drug candidates pose significant analytical and regulatory challenges, requiring rigorous control of interconversion barriers and enantiomeric purity throughout development. The field would benefit from computational tools to predict racemization half-lives and guide the design of configurationally stable candidates.

### 3.3. Cross-Target SAR Decoding

One of the most striking findings is that the same modification positions on the THCz scaffold—notably N-9, C-1, and C-6—exhibit recurrent, transferable SAR trends across phylogenetically and structurally unrelated targets. This phenomenon suggests that these positions govern fundamental molecular recognition properties (hydrophobicity, electronics, conformational mobility) that transcend individual binding sites, enabling the formulation of a cross-target SAR matrix (Table 6) as a practical design guide.

N-9 substitution: Across BTK, AMPK, microtubules, and HDAC6, N-9 acyl or aryl substituents consistently outperform alkyl groups or the unsubstituted parent. This pattern indicates that N-9 functions primarily as a hydrophobic contact point rather than a hydrogen-bond donor/acceptor. The superior performance of electron-withdrawing aroyl groups (e.g., p-chlorobenzoyl in AMPK activator **58** [16]) further implies that modulating the indole nitrogen’s electron density influences π-stacking or dipole interactions with aromatic residues. Notably, N-9 methylation—a common metabolic blocking strategy—is generally tolerated but rarely improves potency, suggesting it should be reserved for late-stage PK optimization.

C-6 substitution: A halogen atom (Br, Cl, I) or trifluoromethyl group at C-6 confers a striking potency advantage in SC inhibitors (**1**) [14], MreB inhibitors (**6**) [26], HPV antiviral agents (2**1**) [46], HDAC6 inhibitors (**35**) [58], Nrf2 modulators (**36**) [61], and 5-HT_6_ antagonists (**68a**–**c**) [92]. The activity hierarchy (Br/Cl > CF_3_ > F > H > EDG) is remarkably consistent. This is unlikely to reflect a universal pharmacophore interaction; rather, halogenation at this position optimizes global molecular properties—increasing lipophilicity, polarizability, and metabolic stability—while enabling context-dependent halogen bonding, hydrophobic packing, or aromatic edge-to-face interactions. The implication is profound: C-6 halogenation should be considered a default optimization step regardless of target, with bromine and chlorine as preferred starting points.

C-1 stereochemistry: The C-1 position is unique among THCz modification sites in exhibiting absolute stereochemical preference across multiple targets (HPV [46], AMPK, pERK/pRb, CRTH2). The consistent superiority of the (*R*)-enantiomer strongly suggests that the C-1 substituent projects into a conserved chiral pocket that recognizes the configuration at this sp^3^ center. This cross-target convergence validates the use of chiral resolution or asymmetric synthesis early in optimization campaigns, rather than treating enantiomers as interchangeable. This matrix can also be used in reverse: given a desired target profile, medicinal chemists can select optimal substituents at each position to accelerate lead optimization.

### 3.4. Precision Intervention

The most recent wave of THCz innovation moves beyond reversible occupation towards precision intervention, such as covalent engagement of otherwise undruggable targets, and spatiotemporally controlled delivery.

Covalent inhibition: The BTK program again provides a compelling case study. Guided by co-crystal structures revealing proximity between the quinazolinone terminus and Cys481, the reversible inhibitor (**54**) [81] was converted into an irreversible acrylamide (**56**) [84] with single-digit picomolar cellular potency. This transformation required no modification of the THCz core itself; rather, an electrophilic warhead was appended via an existing vector, preserving the parent binding mode while achieving kinetic selectivity. This warhead-as-substituent strategy is broadly applicable, provided a suitably positioned nucleophile exists in the target. Encouragingly, the THCz scaffold’s multiple substitution sites offer ample opportunities for vectoral warhead installation without disrupting core recognition.

Targeted photodynamic therapy: The conjugation of N-methylated THCz ligands to the near-infrared photosensitizer IR700DX (**47**) [74] represents a paradigm shift: here, THCz functions not as a pharmacophore but as a targeting vector, directing cytotoxic payloads specifically to TSPO-overexpressing cancer cells. Light-triggered ROS generation induces mitochondrial apoptosis with spatial precision, minimizing systemic toxicity. This approach uncouples the scaffold’s bioactivity from its therapeutic effect—a conceptual leap that decouples affinity from efficacy and opens applications beyond classical enzyme/receptor inhibition.

Prodrug and delivery systems: The click-activated prodrug of (*R*)-**32**, combining trans-cyclooctene conjugation with cRGDyk-Tz-mediated tumor targeting, achieves site-specific activation while sparing normal tissues [55]. Although still nascent, this strategy addresses the long-standing challenge of systemic toxicity for potent THCz antiproliferative agents. It also illustrates a division of labor: the THCz core provides potency, while appended delivery modules govern biodistribution and selectivity.

Covalent THCz inhibitors remain rare, largely because the indole nucleus is inherently weakly electrophilic. Installing reactive warheads without compromising metabolic stability or engendering indiscriminate reactivity requires careful tuning of electrophilicity and linker geometry. For photodynamic and prodrug approaches, key unanswered questions include the optimal ligand–payload distance, the impact of conjugation on TSPO binding kinetics, and the translational feasibility of two-component activation systems.

## 4. Outlook and Perspectives

After more than two decades of research, the THCz scaffold has evolved from a serendipitously discovered hit into a highly versatile platform in drug discovery. Through a systematic analysis of SAR data across diverse therapeutic areas, this review reveals a core conclusion: despite the diversity of targets modulated by THCz derivatives, the SAR trends at key modification sites (N-9, C-1, C-6) exhibit remarkable consistency and transferability across phylogenetically unrelated targets. This cross-target characteristic not only validates the THCz as a genuine “privileged scaffold” but also provides a generalizable logic for future drug design that transcends single-target experience.

Looking forward, we propose three core strategic directions to propel the field from fragmented optimization case studies toward systematic, rational design:

1. From fragmented SAR to a cross-target design logic

The cross-target SAR matrix constructed in this review establishes that C-6 halogenation (particularly Br, Cl) and the stereochemistry at C-1 should be considered “default optimization items” in future THCz molecular design, as they universally enhance lipophilicity, metabolic stability, and fit into hydrophobic pockets. Future research should move beyond isolated explorations of single-site modifications and instead adopt combinatorial optimization strategies (e.g., “C-6 halogen + N-9 aroyl + C-1 chiral amine”) based on this matrix to systematically construct compound libraries, thereby rapidly discovering candidates with multi-target activity profiles.

2. A paradigm shift from target occupancy to precision intervention

The rigid planar structure and multiple modifiable vectors of the THCz scaffold make it an ideal platform for developing drugs with novel mechanisms of action. Future efforts should prioritize the following precision intervention strategies:

Covalent inhibition and targeted protein degradation: Building on the binding modes of existing reversible inhibitors, electrophilic warheads (e.g., acrylamides) can be installed at the C-2 or N-9 positions to target non-catalytic cysteines, developing covalent inhibitors to overcome resistance or prolong target engagement. Concurrently, employing THCz as either an E3 ligase ligand or a target protein ligand to design PROTAC molecules for targeted degradation of pathogenic proteins represents a highly promising new direction.

Theranostic applications: Leveraging the intrinsic fluorescence of THCz or its ease of modification, conjugation with photosensitizers, radionuclides, or fluorophores can yield theranostic probes for tumor imaging or photodynamic therapy.

3. Innovation through the integration of artificial intelligence and chemical biology

With the accumulation of data, the research paradigm for THCz is poised for a transition from experience-driven to data-driven discovery:

AI-guided molecular design: The existing cross-target SAR dataset (>500 compounds) provides an ideal training ground for AI models. Future research should utilize generative AI and machine learning models (e.g., XGBoost, graph neural networks) to explore broader chemical space in silico, designing THCz molecules with customized multi-target activity profiles and favorable pharmacokinetic properties.

Target identification and repurposing: For novel THCz actives identified through phenotypic screening, chemoproteomic techniques should be systematically employed for target deconvolution. This will not only elucidate their mechanisms of action but may also lead to the discovery of entirely new drug targets. Concurrently, profiling existing, well-characterized THCz compound libraries against emerging target families (e.g., senescence, immunomodulation) through affinity-based screens offers an efficient pathway for drug repurposing.

In summary, the full potential of the THCz scaffold remains far from exhausted. Breakthroughs in the next decade will no longer depend on the simple modification of known molecules but on the deep integration of the cross-target SAR principles summarized herein with emerging technologies (AI, PROTAC, chemical biology). This will enable a leap from “discovering a scaffold” to “designing a function,” ultimately transforming THCz into a precision tool for tackling complex diseases.

## Figures and Tables

**Table 2:** Consolidated SAR matrix and design implications for antibacterial THCz derivatives.

Modification Site	Preferred Substituents	Target/ Mechanism	Activity Trend	Design Implication
C-6	Br, Cl, CF_3_ > F > H > EDG	SC, MreB, DNA biosynthesis, CpxRA	Halogen enhances potency; Br/Cl optimal	Default optimization step; halogen bonding or hydrophobic packing
C-2	Carboxylate	SC	(*R*)-enantiomer is preferred	Absolute stereochemical control essential
N-9	Flexible linkers (2–3 atoms)	SC	Reaches subsite II; rigid groups abolish activity	Linker length critical for bivalent engagement
C-1	Amino	CpxRA, MreB	(*R*)-enantiomer is superior	Chiral recognition by target pocket
Side chain	Diamine (protonated)	Cell wall (C_55_-PP)	Electrostatic interaction essential	pH-dependent activity; membrane anchoring
Core size	Cycloheptane-fused > cyclohexane	MreB	Shape complementarity improves potency	Core curvature influences target adaptation
Hybrid design	3-carbon linker optimal	SC/DHFR dual	Synergistic dual-target inhibition	Linker composition and length are critical

**Table 3:** SAR summary of THCz-based Pma1 inhibitors.

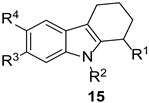								
Compound	R^1^	R^2^	R^3^	R^4^	ATP Hydrolysis IC_50_ [μM]			
					*S. cerevisiae* Pma1	*C. albicans* Pma1	Mammalian SERCA	Mammalian Na^+^, K^+^-ATPase
**15a**	NH-CH_2_-Ph-CI	H	OCF_3_	H	2.79 ± 0.36	3.89 ± 1.71	0.09 ± 0.03	0.72 ± 0.30
**15b**	NH-CH_2_-Ph-Br	H	OCF_3_	H	2.43 ± 0.39	2.47 ± 0.26	0.11 ± 0.06	0.38 ± 0.12
**15c**	NH-CH_2_-CH_2_-O-Ph- CI	H	OCF_3_	H	3.57 ± 1.81	3.77 ± 1.04	0.26 ± 0.06	1.07 ± 0.38
**15d**	NH-CH_2_-2-Phenyl pyridine	H	H	OCF_3_	4.73 ± 2.23	5.56 ± 1.55	3.27 ± 1.89	9.76 ± 4.47
**15e**	NH-CH_2_-Biphenyl	H	H	Cl	6.69 ± 2.06	9.70 ± 2.15	5.54 ± 3.16	5.50 ± 2.74
**15f**	NH-CH_2_-Biphenyl	Me	H	Cl	>20	>20	>20	>20

**Table 4:** Consolidated SAR matrix and design implication for antitumor THCz derivatives.

Modification Site	Preferred Substituents	Target/ Mechanism	Activity Trend	Design Implication
C-6	Halogen (Br, Cl, F) > H; Me, CF_3_, OCF_3_	HDAC6, Nrf2, YAP/TAZ, pERK/pRb, VEGFR-2/EGFR, DNA damage	Halogen (especially Br, Cl) or methyl markedly enhances potency across diverse antitumor targets; electron-withdrawing groups (F, Cl, Br, OCF_3_) generally preferred.	Default optimization step; halogen improves hydrophobic packing, potential halogen bonding, and metabolic stability.
C-1	Amino, amide, heterocycle (thiazole, pyrazole, thiazolidinone); (*R*)-configuration often preferred	VEGFR-2/EGFR, TAM repolarization, TSPO	C-1 serves as a versatile attachment point for pharmacophores; stereochemistry can dictate target selectivity (e.g., (*S*)-enantiomer favors topoisomerases, (*R*)-enantiomer favors EGFR).	Introduce diverse side chains at C-1; resolve enantiomers early to exploit chiral recognition and fine-tune selectivity.
C-2	Mannich base (dialkylamino, piperidinyl), amino, benzylamino	Microtubule, pERK/pRb	Basic amines at C-2 significantly improve potency; bulky aryl groups (e.g., benzyl) enhance pERK/pRb inhibition.	Install cationic centers for electrostatic interactions with target pockets; balance lipophilicity and basicity.
C-3	Thioamide, amide, spirooxindole	DNA damage, mitochondrial dysfunction	Thioamide > amide for potency; synergy with C-6 electron-withdrawing groups; spirooxindole fusion induces ROS-mediated apoptosis.	Consider thioamide as a bioisostere of amide to boost binding; spirocyclic scaffolds offer novel mechanisms.
N-9	Methyl, acyl (amide/urea), aryl (sulfonyl, benzoyl), tricyclic cap	Microtubule, HDAC6, TSPO, pERK/pRb	N-9 methylation enhances microtubule inhibition; N-9 amide/urea essential for certain microtubule agents; tricyclic cap optimizes HDAC6 engagement.	Modify N-9 to modulate lipophilicity, π-stacking, and metabolic stability; cyclization or bulky caps can lock bioactive conformations.
Side chain/Linker	3-carbon linker optimal for hybrids; aromatic/heteroaromatic spacers	Multi-kinase hybrids, TAM repolarization, DNA damage	Linker length and composition critically affect dual-target synergy; heteroaromatic linkers enhance DNA interaction.	Optimize linker rigidity and length to balance entropic penalty and simultaneous target engagement; explore bioisosteric replacements.

**Table 5:** Representative THCz-based compounds: structures, targets, activities, and development status.

Compound	Scaffold Core	Therapeutic Area	Target(s)	Activity (IC_50_/*K*ᵢ/MIC)	Development Stage	Ref.
**2a**	6-Chloro-THCz	Antibacterial	*E. coli* sliding clamp	*K*ᵢ = 20 μM	Hit	[20]
**4**	THCz-2,4-diaminopyrimidine	Antibacterial	Bacterial sliding clamp/DHFR	MIC = 0.39–0.78 μg/mL (MRSA)	Hit	[22]
**6**	THCz-cyclohexylamine	Antibacterial	MreB	MIC = 1 μg/mL (*P. aeruginosa* efflux-deficient)	Lead	[26]
**23** (GSK983)	1-Amino-THCz	Antiviral (HPV)	HPV (host-targeting)	IC_50_ = 0.03 μM (W12 cell)	Lead	[46]
**32**	3-Amino-THCz	Antitumor	Targeting DNA damage and oxidative stress	IC_50_ = 45 nM (multiple cancer cell lines)	Lead	[55]
**35**	6-Bromine-THCz	Antitumor	HDAC6	IC_50_ = 0.51–0.52 μM (SUNE1, MDA-MB-231)	Lead	[58]
**47**	N-Methyl-THCz	Antitumor	TSPO (photosensitizer conjugate)	*K*ᵢ = 155 nM	Lead	[74]
**54**	THCz (quinazolinone-dione)	Autoimmune (RA)	BTK	IC_50_ = 0.50 nM	Phase II	[81]
**56**	THCz-acrylamide	Autoimmune (RA)	BTK (covalent)	IC_50_ = 0.21 nM	Lead	[84]
**58**	6-benzyloxy-THCz	T2DM	AMPK	Glucose uptake increases 82.5% (HepG2)	Lead	[14]
**62**	THCz-benzylpyridine	Alzheimer’s disease	BuChE	IC_50_ = 0.088 μM	Lead	[16]
**63**	Aminoadamantane-THCz	Alzheimer’s disease	BuChE/MCU	IC_50_ = 5.43 μM	Lead	[89]
**68b**	6-OCH_3_-THCz	Alzheimer’s disease	5-HT_6_	*K*ᵢ = 5.44 nM; IC_50_ = 457 nM	Lead	[92]
**71**	5-Fluoro-4-azaindole (THCz bioisostere)	Allergic inflammation	CRTH2	IC_50_ = 4 nM (*h*CRTH2)	Preclinical candidate	[97]

**Table 6:** Cross-target SAR matrix of the THCz scaffold.

Modification Site	Preferred Substituents	Design Implication	Representative Targets	Representative Compounds	Key Refs.
N-9	Acyl, aryl, benzoyl > alkyl > H	Hydrophobic contact, π–stacking, electronic modulation; cyclization can lock conformation and improve metabolic stability	BTK, AMPK, Microtubule, HDAC6, CRTH2	**26**, **35**, **58**, **62**, **64**	[14,16,51,58,92]
C-1	(*R*)-amine, (*R*)-benzylamine, (*R*)-amide	Stereospecific hydrogen bond/ionic interaction; vector projection into a conserved chiral pocket	HPV, pERK/pRb, Pma1, TSPO, CRTH2, AMPK	**11**, **13**, **21**, **30**, **39**	[30,36,46,54,68]
C-6	Br, Cl, CF_3_ > F > H > EDG	Halogen bonding, hydrophobic packing, metabolic stabilization; enhances lipophilicity and polarizability	SC, MreB, HPV, HDAC6, Nrf2, 5-HT_6_	**1**, **6**, **36**, **54**	[14,26,61,81]
C-2/C-3	Carboxylate, Mannich base, thioamide, electrophilic warhead (acrylamide)	Polar contact, metal chelation, covalent modification; provides additional binding energy or covalent reactivity	SC, Microtubule, Topoisomerase	**2**, **32**, **44**, **46**	[20,55,70,72]
C-5/C-8	Disubstituted (e.g., 5,8-Cl_2_, 5-CN-8-OCH_3_)	Fills hydrophobic subpocket; disubstitution synergistically enhances binding affinity	HCV NS5B	**20**	[44]
C-7	F, Cl, CF_3_O	Modulates electron density, improves binding free energy; site-preferential halogenation	Pma1, AMPK	**15b**, **59**	[15,41]
Ring Size	Cyclopentane-, cyclohexane-, cycloheptane-fused indole	Adjusts core curvature and shape complementarity; influences solubility, toxicity, target adaptation	MreB	**8**, **9**	[12]
Conformational Locking	Control atropisomerism; side-chain cyclization	Restricts rotational freedom, reduces entropic penalty; improves selectivity and whole-blood activity	BTK	**54**, **55**, **68**	[80,81,82]

## Data Availability

No new data were created or analyzed in this study.
